# Supplementary material for: GWAS and PheWAS of red blood cell components in a Northern Nevadan cohort
Source: PLoS One. 2019 Jun 13;14(6):e0218078. doi: 10.1371/journal.pone.0218078 (PMC6564422; doi:10.1371/journal.pone.0218078)
Supplement: S1 Table — This table includes mean standardized RBC component values for each individual along with age and gender. Due to the length of this table it can be found online at www.dri.edu/HealthyNVProjectGenetics. (PDF) [file pone.0218078.s001.pdf]

Supplementary Table 1: Due to the length of this table, it can be found online at www.dri.edu/HealthyNVProjectGenetics
