# Supplementary material for: GWAS and PheWAS of red blood cell components in a Northern Nevadan cohort
Source: PLoS One. 2019 Jun 13;14(6):e0218078. doi: 10.1371/journal.pone.0218078 (PMC6564422; doi:10.1371/journal.pone.0218078)
Supplement: S2 Table — This table lists the 38 statistically significant SNPs associated to MPV, MCV and PC in our cohort. General information about the SNP such as chromosome location, GWAS p-value, power, genotype, cytoband, ANOVA, and references of associations identified in previous studies are listed. (PDF) [file pone.0218078.s002.pdf]

rsID	N	GWAS *p*-value	power of GWAS	Genotype 1	Genotype 2	Genotype 3	Mean +/- SD Genotype 1	Mean +/- SD Genotype 2	Mean +/- SD Genotype 3	ANOVA *p*-value	Chromosome	Cytoband Region	SNP Previously Identified	Cytoband Region Referenced	RBC Component
rs10274553	4562	3.82x10^{-9}	>90	T/T	T/C	C/C	10.72 +/- 0.99	10.57 +/- 0.98	10.46 +/- 0.94	4.50x10^{-10}	chr3	p14.3	[13,18,28,33]	[18,28]	MPV
rs10509186	4562	7.75x10^{-9}	>90	C/C	C/T	T/T	10.69 +/- 1	10.56 +/- 0.97	10.47 +/- 0.95	2.61x10^{-7}	chr3	p14.3	N/A	[18,28]	MPV
rs10822186	4564	4.38x10^{-8}	>90	A/A	A/G	G/G	10.69 +/- 0.99	10.57 +/- 0.97	10.49 +/- 0.96	3.13x10^{-6}	chr7	q22.3	[13,17,25,28,30,35	[13,25,59]	MPV
rs11130549	4562	9.94x10^{-9}	>90	T/T	T/C	C/C	10.66 +/- 0.99	10.55 +/- 0.97	10.44 +/- 0.93	2.22x10^{-6}	chr7	q22.3	[35]	[13,25,59]	MPV
rs12355784	4551	9.32x10^{-9}	>90	C/C	C/A	A/A	10.69 +/- 1	10.56 +/- 0.97	10.48 +/- 0.96	7.42x10^{-7}	chr7	q22.3	[13,28]	[13,25,59]	MPV
rs1354034	4556	2.39x10^{-13}	>90	C/C	C/T	T/T	10.46 +/- 0.94	10.6 +/- 0.97	10.79 +/- 1.03	2.40x10^{-13}	chr7	q22.3	[18,30]	[13,25,59]	MPV
rs1788103	4564	5.15x10^{-10}	>90	A/A	A/G	G/G	10.69 +/- 1.01	10.58 +/- 0.97	10.45 +/- 0.95	2.59x10^{-8}	chr7	q22.3	N/A	[13,25,59]	MPV
rs1790588	4563	3.31x10^{-10}	>90	T/T	T/C	C/C	10.69 +/- 1.01	10.58 +/- 0.97	10.45 +/- 0.94	1.70x10^{-8}	chr7	q22.3	N/A	[13,25,59]	MPV
rs1790974	4563	3.32x10^{-8}	>90	C/C	C/T	T/T	10.67 +/- 1.02	10.57 +/- 0.96	10.46 +/- 0.93	1.14x10^{-6}	chr10	q21.3	N/A	[18,30,49]	MPV
rs1935	4558	3.57x10^{-8}	>90	C/C	C/G	G/G	10.68 +/- 1	10.57 +/- 0.97	10.48 +/- 0.95	2.53x10^{-6}	chr10	q21.3	[49,50]	[18,30,49]	MPV
rs201979226	4551	5.89x10^{-9}	>90	T/T	T/C	C/C	10.47 +/- 0.93	10.57 +/- 0.99	10.73 +/- 0.99	4.82x10^{-10}	chr10	q21.3	[51,52,53]	[18,30,49]	MPV
rs342240	4563	3.49x10^{-10}	>90	G/G	G/A	A/A	10.48 +/- 0.95	10.59 +/- 0.98	10.76 +/- 1	8.26x10^{-11}	chr10	q21.3	[18,33]	[18,30,49]	MPV
rs342275	4564	2.96x10^{-10}	>90	C/C	C/T	T/T	10.48 +/- 0.94	10.59 +/- 0.98	10.77 +/- 1	6.45x10^{-11}	chr10	q21.3	[54,55]	[18,30,49]	MPV
rs342293	4559	6.61x10^{-11}	>90	C/C	C/G	G/G	10.48 +/- 0.93	10.57 +/- 0.99	10.77 +/- 0.99	5.44x10^{-12}	chr10	q21.3	[35,56]	[18,30,49]	MPV
rs342296	4561	1.04x10^{-10}	>90	G/G	G/A	A/A	10.47 +/- 0.93	10.58 +/- 0.99	10.77 +/- 0.98	1.26x10^{-11}	chr10	q21.3	[53]	[18,30,49]	MPV
rs34818942	4555	7.77x10^{-11}	>90	C/C	C/T	T/T	10.55 +/- 0.97	10.79 +/- 1.01	11.26 +/- 0.67	7.90x10^{-11}	chr12	q24.31	[13,18,24,28]	[18,60]	MPV
rs386614085	4564	1.21x10^{-8}	>90	A/A	A/G	G/G	10.69 +/- 1	10.56 +/- 0.97	10.48 +/- 0.96	9.58x10^{-7}	chr12	q24.31	N/A	[18,60]	MPV
rs4379723	4555	1.29x10^{-8}	>90	T/T	T/C	C/C	10.68 +/- 1	10.57 +/- 0.97	10.47 +/- 0.95	1.01x10^{-6}	chr18	q22.2	[57]	[17]	MPV
rs763361	4564	3.26x10^{-10}	>90	C/C	C/T	T/T	10.7 +/- 1.01	10.58 +/- 0.97	10.45 +/- 0.94	1.48x10^{-8}	chr18	q22.2	[58]	[17]	MPV
rs7910927	4548	2.68x10^{-8}	>90	T/T	T/G	G/G	10.68 +/- 1	10.56 +/- 0.97	10.48 +/- 0.95	1.74x10^{-6}	chr18	q22.2	N/A	[17]	MPV
rs7961894	4564	2.68x10^{-11}	>90	C/C	C/T	T/T	10.54 +/- 0.97	10.74 +/- 1	11.21 +/- 0.87	8.70x10^{-12}	chr18	q22.2	N/A	[17]	MPV
rs218237	4672	5.07x10^{-9}	>90%	C/C	C/T	T/T	91.4 +/- 4.47	91.84 +/- 4.41	91.92 +/- 4.86	8.40x10^{-3}	chr4	q12	[45]	[20,27,32]	MCV
rs9402686	4670	4.60x10x^{-10}	>90%	G/G	G/A	A/A	91.13 +/- 4.4	91.94 +/- 4.46	92.65 +/- 4.76	1.88x10^{-12}	chr6	q23.3	[17,27,40]	[20,27,32]	MCV
rs7776054	4672	6.65x10^{-10}	>90%	A/A	A/G	G/G	91.17 +/- 4.41	91.88 +/- 4.48	92.81 +/- 4.54	7.85x10^{-12}	chr6	q23.3	[21,23,39,42,44]	[20,27,32]	MCV
rs9399137	4667	7.82x10^{-10}	>90%	T/T	T/C	C/C	91.15 +/- 4.45	91.93 +/- 4.42	92.81 +/- 4.53	9.76x10^{-13}	chr6	q23.3	[20,21,28,42]	[20,27,32]	MCV
rs7775698	4669	8.24x10^{-10}	>90%	C/C	C/T	T/T	91.17 +/- 4.41	91.88 +/- 4.48	92.79 +/- 4.55	1.12x10^{-11}	chr6	q23.3	[26,27,33]	[20,27,32]	MCV
rs4895441	4672	1.27x10^{-9}	>90%	A/A	A/G	G/G	91.13 +/- 4.4	91.94 +/- 4.45	92.57 +/- 4.76	5.77x10^{-12}	chr6	q23.3	[20,23,27,37]	[20,27,32]	MCV
rs111194878	4670	1.47x10^{-9}	>90%	C/C	C/A	A/A	91.15 +/- 4.4	91.89 +/- 4.46	92.61 +/- 4.77	4.13x10^{-11}	chr6	q23.3	[20,27,41]	[20,27,32]	MCV
rs9373124	4654	5.14x10^{-9}	>90%	T/T	T/C	C/C	91.19 +/- 4.36	91.84 +/- 4.51	92.49 +/- 4.91	8.46x10^{-9}	chr6	q23.3	[29,43]	[20,27,32]	MCV
rs855791	4670	5.23x10^{-12}	>90%	G/G	G/A	A/A	92.09 +/- 4.44	91.47 +/- 4.48	90.77 +/- 4.36	1.60x10^{-11}	chr22	q12.3	[27,31,33,36,38]	[27,32]	MCV
rs4820268	4616	2.65x10^{-11}	>90%	A/A	A/G	G/G	92.08 +/- 4.53	91.54 +/- 4.44	90.76 +/- 4.4	2.03x10^{-11}	chr22	q12.3	[22,27,31]	[27,32]	MCV
rs5756504	4672	7.77x10^{-10}	>90%	C/C	C/T	T/T	91.03 +/- 4.38	91.75 +/- 4.47	92.19 +/- 4.57	5.02x10^{-10}	chr22	q12.3	[26,27,45]	[27,32]	MCV
rs130624	4654	1.13x10^{-9}	>90%	T/T	T/G	G/G	91.01 +/- 4.28	91.65 +/- 4.58	92.14 +/- 4.42	3.27x10^{-9}	chr22	q12.3	[32]	[27,32]	MCV
rs5756506	4591	1.15x10^{-9}	>90%	G/G	G/C	C/C	91.05 +/- 4.38	91.74 +/- 4.5	92.18 +/- 4.55	1.77x10^{-9}	chr22	q12.3	[17,27]	[27,32]	MCV
rs386563505	4671	7.12x10^{-9}	>90%	G/G	G/A	A/A	91.09 +/- 4.35	91.62 +/- 4.53	92.16 +/- 4.46	6.54x10^{-8}	chr22	q12.3	N/A	[27,32]	MCV
rs385893	4672	8.04x10^{-10}	>90%	C/C	C/T	T/T	260.83 +/- 65.81	250.23 +/- 61.86	244.26 +/- 57.71	2.58x10^{-10}	chr9	p24.1	[17,25,26,34]	[17,18,25]	PC
rs10974808	4664	3.53x10^{-9}	>90%	A/A	A/G	G/G	249.15 +/- 61.55	259.41 +/- 62.62	271.78 +/- 76.72	7.39x10^{-7}	chr9	p24.1	N/A	[17,18,25]	PC
rs423955	4672	2.64x10^{-8}	>90%	T/T	T/C	C/C	256.85 +/- 62.51	249.14 +/- 63.06	241.53 +/- 55.74	1.12x10^{-7}	chr9	p24.1	[18]	[17,18,25]	PC
