# Supplementary material for: GWAS and PheWAS of red blood cell components in a Northern Nevadan cohort
Source: PLoS One. 2019 Jun 13;14(6):e0218078. doi: 10.1371/journal.pone.0218078 (PMC6564422; doi:10.1371/journal.pone.0218078)
Supplement: S3 Table — This table presents the mapping between ICD9 codes and phecodes as presented in Carroll and the R package PheWAS [86] tested in our study, and the number of incidences from the RBC cohort in each phecode group. (PDF) [file pone.0218078.s003.pdf]

Phecode	Description	Phenotype Group	Phenotype Group Description	PC Counts	MPV Counts	MCV Counts
8	Intestinal infection	1	infectious diseases	3	3	3
8.5	Bacterial enteritis	1	infectious diseases	0	0	0
8.51	Intestinal e.coli	1	infectious diseases	0	0	0
8.52	Intestinal infection due to C. difficile	1	infectious diseases	0	0	0
8.6	Viral Enteritis	1	infectious diseases	0	0	0
8.7	Intestinal infection due to protozoa	1	infectious diseases	0	0	0
10	Tuberculosis	1	infectious diseases	1	1	1
31	Diseases due to other mycobacteria	1	infectious diseases	0	0	0
31.1	Leprosy	1	infectious diseases	0	0	0
38	Septicemia	1	infectious diseases	32	32	32
38.1	Gram negative septicemia	1	infectious diseases	0	0	0
38.2	Gram positive septicemia	1	infectious diseases	1	1	1
38.3	Bacteremia	1	infectious diseases	2	2	2
41	Bacterial infection NOS	1	infectious diseases	219	219	219
41.1	Staphylococcus infections	1	infectious diseases	24	24	24
41.11	Methicillin sensitive Staphylococcus aureus	1	infectious diseases	1	1	1
41.12	Methicillin resistant Staphylococcus aureus	1	infectious diseases	14	14	14
41.2	Streptococcus infection	1	infectious diseases	16	16	16
41.21	Rheumatic fever / chorea	1	infectious diseases	3	3	3
41.4	E. coli	1	infectious diseases	22	22	22
41.8	H. pylori	1	infectious diseases	6	6	6
41.9	Infection with drug-resistant microorganisms	1	infectious diseases	4	4	4
53	Herpes zoster	1	infectious diseases	0	0	0
53.1	Herpes zoster with nervous system complications	1	infectious diseases	0	0	0
54	Herpes simplex	1	infectious diseases	7	7	7
70	Viral hepatitis	1	infectious diseases	6	6	6
70.1	Viral hepatitis A	1	infectious diseases	0	0	0
70.2	Viral hepatitis B	1	infectious diseases	1	1	1
70.3	Viral hepatitis C	1	infectious diseases	4	4	4
70.4	Chronic hepatitis	1	infectious diseases	6	6	6
70.9	Hepatitis NOS	1	infectious diseases	7	7	7
71	Human immunodeficiency virus [HIV] disease	1	infectious diseases	8	8	8
71.1	HIV infection, symptomatic	1	infectious diseases	1	1	1
78	Viral warts & HPV	1	infectious diseases	10	10	10
79	Viral infection	1	infectious diseases	22	22	22
79.1	Varicella infection	1	infectious diseases	0	0	0
79.2	Infectious mononucleosis	1	infectious diseases	3	3	3
79.9	Viremia, NOS	1	infectious diseases	1	1	1
80	Postoperative infection	1	infectious diseases	17	17	17
81	Infection/inflammation of internal prosthetic device; implant; and graft	1	infectious diseases	9	9	9
81.1	Graft-versus-host disease	1	infectious diseases	0	0	0
81.11	Acute graft-versus-host disease	1	infectious diseases	0	0	0
81.12	Chronic graft-versus-host disease	1	infectious diseases	0	0	0
90	Sexually transmitted infections (not HIV or hepatitis)	1	infectious diseases	1	1	1
90.2	Gonococcal infections	1	infectious diseases	0	0	0
90.3	Venereal diseases due to Chlamydia trachomatis	1	infectious diseases	0	0	0
110	Dermatophytosis / Dermatomycosis	1	infectious diseases	122	122	122
110.1	Dermatophytosis	1	infectious diseases	104	104	104
110.11	Dermatophytosis of nail	1	infectious diseases	57	57	57
110.12	Althete's foot	1	infectious diseases	13	13	13
110.13	Dermatophytosis of the body	1	infectious diseases	24	24	24
110.2	Dermatomycoses	1	infectious diseases	14	14	14
112	Candidiasis	1	infectious diseases	139	139	139
112.3	Candidiasis of skin and nails	1	infectious diseases	22	22	22
117	Mycoses	1	infectious diseases	9	9	9
117.1	Histoplasmosis	1	infectious diseases	0	0	0
117.2	Coccidioidomycosis	1	infectious diseases	3	3	3
117.3	Blastomycotic infection	1	infectious diseases	0	0	0
117.4	Aspergillosis	1	infectious diseases	0	0	0
130	Spirochetal infection	1	infectious diseases	0	0	0
130.1	Lyme disease	1	infectious diseases	0	0	0
131	Protozoan infection	1	infectious diseases	3	3	3
132	Infestation (lice, mites)	1	infectious diseases	5	5	5
132.1	Pediculosis and phthirus infestation	1	infectious diseases	1	1	1
133	Arthropod-borne diseases	1	infectious diseases	0	0	0

134	Helminthiases	1	infectious diseases	0	0	0
134.1	Intestinal helminthiases	1	infectious diseases	0	0	0
136	Other infectious and parasitic diseases	1	infectious diseases	33	33	33
145	Cancer of mouth	2	neoplasms	1	1	1
145.1	Cancer of lip	2	neoplasms	0	0	0
145.2	Cancer of tongue	2	neoplasms	1	1	1
145.3	Cancer of major salivary glands	2	neoplasms	0	0	0
145.4	Cancer of the gums	2	neoplasms	0	0	0
145.5	Cancer of the mouth floor	2	neoplasms	0	0	0
149	Cancer of larynx, pharynx, nasal cavities	2	neoplasms	0	0	0
149.1	Cancer of oropharynx	2	neoplasms	0	0	0
149.2	Cancer of nasopharynx	2	neoplasms	0	0	0
149.3	Cancer of hypopharynx	2	neoplasms	0	0	0
149.4	Cancer of larynx	2	neoplasms	0	0	0
149.5	Hx of malignant neoplasm of oral cavity and pharynx	2	neoplasms	0	0	0
149.9	Cancer of of nasal cavities	2	neoplasms	0	0	0
150	Cancer of esophagus	2	neoplasms	2	2	2
151	Cancer of stomach	2	neoplasms	1	1	1
153	Colorectal cancer	2	neoplasms	25	25	25
153.2	Colon cancer	2	neoplasms	22	22	22
153.3	Malignant neoplasm of rectum, rectosigmoid junction, and anus	2	neoplasms	7	7	7
155	Cancer of liver and intrahepatic bile duct	2	neoplasms	5	5	5
155.1	Malignant neoplasm of liver, primary	2	neoplasms	2	2	2
157	Pancreatic cancer	2	neoplasms	4	4	4
158	Neoplasm of unspecified nature of digestive system	2	neoplasms	4	4	4
159	Malignant neoplasm of other and ill-defined sites within the digestive organs and peritoneum	2	neoplasms	6	6	6
159.2	Malignant neoplasm of small intestine, including duodenum	2	neoplasms	1	1	1
159.3	Malignant neoplasm of gallbladder and extrahepatic bile ducts	2	neoplasms	1	1	1
159.4	Malignant neoplasm of retroperitoneum and peritoneum	2	neoplasms	0	0	0
164	Cancer of intrathoracic organs	2	neoplasms	0	0	0
165	Cancer within the respiratory system	2	neoplasms	11	11	11
165.1	Cancer of bronchus; lung	2	neoplasms	10	10	10
170	Cancer of bone and connective tissue	2	neoplasms	4	4	4
170.1	Bone cancer	2	neoplasms	0	0	0
170.2	Cancer of connective tissue	2	neoplasms	3	3	3
172	Skin cancer	2	neoplasms	145	145	145
172.1	Melanomas of skin, dx or hx	2	neoplasms	34	34	34
172.11	Melanomas of skin	2	neoplasms	18	18	18
172.2	Other non-epithelial cancer of skin	2	neoplasms	109	109	109
172.21	Basal cell carcinoma (new)	2	neoplasms	29	29	29
172.22	Squamous cell carcinoma	2	neoplasms	5	5	5
172.3	Carcinoma in situ of skin	2	neoplasms	1	1	1
173	Neoplasm of uncertain behavior of skin	2	neoplasms	35	35	35
174	Breast cancer	2	neoplasms	157	157	157
174.1	Breast cancer [female]	2	neoplasms	155	155	155
174.11	Malignant neoplasm of female breast	2	neoplasms	155	155	155
174.2	Breast cancer [male]	2	neoplasms	0	0	0
174.3	Neoplasm of uncertain behavior of breast	2	neoplasms	1	1	1
175	Acquired absence of breast	2	neoplasms	37	37	37
180	Cervical cancer and dysplasia	2	neoplasms	20	20	20
180.1	Cervical cancer	2	neoplasms	10	10	10
180.3	Cervical intraepithelial neoplasia [CIN] [Cervical dysplasia]	2	neoplasms	10	10	10
182	Malignant neoplasm of uterus	2	neoplasms	18	18	18
184	Cancer of other female genital organs	2	neoplasms	13	13	13
184.1	Malignant neoplasm of ovary and other uterine adnexa	2	neoplasms	10	10	10
184.11	Malignant neoplasm of ovary	2	neoplasms	9	9	9
184.2	Cancer of other female genital organs	2	neoplasms	2	2	2
185	Cancer of prostate	2	neoplasms	52	52	52
187	Cancer of other male genital organs	2	neoplasms	3	3	3
187.1	Malignant neoplasm of unspecified male genital organ	2	neoplasms	1	1	1
187.2	Malignant neoplasm of testis	2	neoplasms	2	2	2
187.8	Neoplasm of uncertain behavior of male genital organs	2	neoplasms	0	0	0
189	Cancer of urinary organs (incl. kidney and bladder)	2	neoplasms	27	27	27
189.1	Cancer of kidney and renal pelvis	2	neoplasms	17	17	17
189.11	Malignant neoplasm of kidney, except pelvis	2	neoplasms	4	4	4
189.12	Malignant neoplasm of renal pelvis	2	neoplasms	1	1	1
189.2	Cancer of bladder	2	neoplasms	9	9	9

189.21	Malignant neoplasm of bladder	2	neoplasms	7	7	7
189.4	Malignant neoplasm of other urinary organs	2	neoplasms	9	9	9
190	Cancer of eye	2	neoplasms	3	3	3
191	Malignant and unknown neoplasms of brain and nervous system	2	neoplasms	11	11	11
191.1	Cancer of brain and nervous system	2	neoplasms	5	5	5
191.11	Cancer of brain	2	neoplasms	4	4	4
193	Thyroid cancer	2	neoplasms	20	20	20
194	Cancer of other endocrine glands	2	neoplasms	0	0	0
195	Cancer, suspected or other	2	neoplasms	64	64	64
195.1	Malignant neoplasm, other	2	neoplasms	59	59	59
196	Radiotherapy	2	neoplasms	0	0	0
197	Chemotherapy	2	neoplasms	29	29	29
198	Secondary malignant neoplasm	2	neoplasms	26	26	26
198.1	Secondary malignancy of lymph nodes	2	neoplasms	5	5	5
198.2	Secondary malignancy of respiratory organs	2	neoplasms	5	5	5
198.3	Secondary malignant neoplasm of digestive systems	2	neoplasms	0	0	0
198.4	Secondary malignant neoplasm of liver	2	neoplasms	5	5	5
198.5	Secondary malignancy of brain/spine	2	neoplasms	2	2	2
198.6	Secondary malignancy of bone	2	neoplasms	10	10	10
198.7	Secondary malignant neoplasm of skin	2	neoplasms	0	0	0
199	Neoplasm of uncertain behavior	2	neoplasms	94	94	94
199.4	Neurofibromatosis	2	neoplasms	2	2	2
200	Myeloproliferative disease	2	neoplasms	21	21	21
200.1	Polycythemia vera	2	neoplasms	8	8	8
201	Hodgkin's disease	2	neoplasms	6	6	6
202	Cancer of other lymphoid, histiocytic tissue	2	neoplasms	29	29	29
202.2	Non-Hodgkins lymphoma	2	neoplasms	26	26	26
202.21	Nodular lymphoma	2	neoplasms	5	5	5
202.22	Reticulosarcoma	2	neoplasms	0	0	0
202.23	Lymphosarcoma	2	neoplasms	0	0	0
202.24	Large cell lymphoma	2	neoplasms	1	1	1
204	Leukemia	2	neoplasms	14	14	14
204.1	Lymphoid leukemia	2	neoplasms	7	7	7
204.11	Lymphoid leukemia, acute	2	neoplasms	1	1	1
204.12	Lymphoid leukemia, chronic	2	neoplasms	6	6	6
204.2	Myeloid leukemia	2	neoplasms	0	0	0
204.21	Myeloid leukemia, acute	2	neoplasms	0	0	0
204.22	Myeloid leukemia, chronic	2	neoplasms	0	0	0
204.3	Monocytic leukemia	2	neoplasms	0	0	0
204.4	Multiple myeloma	2	neoplasms	5	5	5
208	Benign neoplasm of colon	2	neoplasms	149	149	149
209	Neuroendocrine tumors	2	neoplasms	4	4	4
210	Benign neoplasm of lip, oral cavity, and pharynx	2	neoplasms	1	1	1
211	Benign neoplasm of other parts of digestive system	2	neoplasms	4	4	4
212	Benign neoplasm of respiratory and intrathoracic organs	2	neoplasms	1	1	1
213	Benign neoplasm of bone and articular cartilage	2	neoplasms	1	1	1
214	Lipoma	2	neoplasms	47	47	47
214.1	Lipoma of skin and subcutaneous tissue	2	neoplasms	17	17	17
215	Other benign neoplasm of connective and other soft tissue	2	neoplasms	10	10	10
216	Benign neoplasm of skin	2	neoplasms	65	65	65
216.1	Screening for malignant neoplasms of the skin	2	neoplasms	39	39	39
217	Vascular hamartomas and non-neoplastic nevi	2	neoplasms	11	11	11
217.1	Nevus, non-neoplastic	2	neoplasms	11	11	11
218	Benign neoplasm of uterus	2	neoplasms	64	64	64
218.1	Uterine leiomyoma	2	neoplasms	62	62	62
218.2	Other benign neoplasm of uterus	2	neoplasms	0	0	0
220	Benign neoplasm of ovary	2	neoplasms	4	4	4
221	Benign neoplasm of other female genital organs	2	neoplasms	0	0	0
222	Benign neoplasm of male genital organs	2	neoplasms	0	0	0
223	Benign neoplasm of kidney and other urinary organs	2	neoplasms	3	3	3
224	Benign neoplasm of eye	2	neoplasms	3	3	3
224.1	Benign neoplasm of eye, uveal	2	neoplasms	1	1	1
225	Benign neoplasm of brain and other parts of nervous system	2	neoplasms	22	22	22
225.1	Benign neoplasm of brain, cranial nerves, meninges	2	neoplasms	18	18	18
225.2	Benign neoplasm of spinal cord, meninges	2	neoplasms	0	0	0
226	Benign neoplasm of thyroid glands	2	neoplasms	1	1	1
227	Benign neoplasm of other endocrine glands and related structures	2	neoplasms	31	31	31

227.1	Benign neoplasm of adrenal gland	2	neoplasms	2	2	2
227.2	Benign neoplasm of parathyroid gland	2	neoplasms	2	2	2
227.3	Benign neoplasm of pituitary gland and craniopharyngeal duct (pouch)	2	neoplasms	20	20	20
228	Hemangioma and lymphangioma, any site	2	neoplasms	18	18	18
228.1	Hemangioma of skin and subcutaneous tissue	2	neoplasms	1	1	1
229	Benign neoplasm of unspecified sites	2	neoplasms	6	6	6
229.1	Benign neoplasm of lymph nodes	2	neoplasms	0	0	0
240	Simple and unspecified goiter	3	endocrine/metabolic	42	42	42
241	Nontoxic nodular goiter	3	endocrine/metabolic	160	160	160
241.1	Nontoxic uninodular goiter	3	endocrine/metabolic	0	0	0
241.2	Nontoxic multinodular goiter	3	endocrine/metabolic	79	79	79
242	Thyrotoxicosis with or without goiter	3	endocrine/metabolic	59	59	59
242.1	Graves' disease	3	endocrine/metabolic	5	5	5
242.2	Toxic multinodular goiter	3	endocrine/metabolic	1	1	1
242.3	Exophthalmos	3	endocrine/metabolic	7	7	7
242.31	Thyrotoxic exophthalmos	3	endocrine/metabolic	4	4	4
244	Hypothyroidism	3	endocrine/metabolic	803	803	803
244.1	Secondary hypothyroidism	3	endocrine/metabolic	8	8	8
244.2	Acquired hypothyroidism	3	endocrine/metabolic	249	249	249
244.3	Iodine hypothyroidism	3	endocrine/metabolic	0	0	0
244.4	Hypothyroidism NOS	3	endocrine/metabolic	732	732	732
244.5	Congenital hypothyroidism	3	endocrine/metabolic	11	11	11
245	Thyroiditis	3	endocrine/metabolic	100	100	100
245.1	Thyroiditis, acute and subacute	3	endocrine/metabolic	1	1	1
245.2	Chronic thyroiditis	3	endocrine/metabolic	89	89	89
245.21	Chronic lymphocytic thyroiditis	3	endocrine/metabolic	88	88	88
246	Other disorders of thyroid	3	endocrine/metabolic	241	241	241
246.2	Thyroid cyst	3	endocrine/metabolic	4	4	4
246.7	Abnormal results of function study of thyroid	3	endocrine/metabolic	90	90	90
249	Secondary diabetes mellitus	3	endocrine/metabolic	13	13	13
250	Diabetes mellitus	3	endocrine/metabolic	411	411	411
250.1	Type 1 diabetes	3	endocrine/metabolic	36	36	36
250.11	Type 1 diabetes with ketoacidosis	3	endocrine/metabolic	3	3	3
250.12	Type 1 diabetes with renal manifestations	3	endocrine/metabolic	6	6	6
250.13	Type 1 diabetes with ophthalmic manifestations	3	endocrine/metabolic	3	3	3
250.14	Type 1 diabetes with neurological manifestations	3	endocrine/metabolic	5	5	5
250.15	Diabetes type 1 with peripheral circulatory disorders	3	endocrine/metabolic	0	0	0
250.2	Type 2 diabetes	3	endocrine/metabolic	218	218	218
250.21	Type 2 diabetes with ketoacidosis	3	endocrine/metabolic	2	2	2
250.22	Type 2 diabetes with renal manifestations	3	endocrine/metabolic	31	31	31
250.23	Type 2 diabetes with ophthalmic manifestations	3	endocrine/metabolic	12	12	12
250.24	Type 2 diabetes with neurological manifestations	3	endocrine/metabolic	21	21	21
250.25	Diabetes type 2 with peripheral circulatory disorders	3	endocrine/metabolic	7	7	7
250.3	Insulin pump user	3	endocrine/metabolic	73	73	73
250.4	Abnormal glucose	3	endocrine/metabolic	661	661	661
250.41	Impaired fasting glucose	3	endocrine/metabolic	402	402	402
250.42	Other abnormal glucose	3	endocrine/metabolic	337	337	337
250.5	Glycosuria or Acetonuria	3	endocrine/metabolic	6	6	6
250.6	Polyneuropathy in diabetes	3	endocrine/metabolic	26	26	26
250.7	Diabetic retinopathy	3	endocrine/metabolic	11	11	11
251	Other disorders of pancreatic internal secretion	3	endocrine/metabolic	1	1	1
251.1	Hypoglycemia	3	endocrine/metabolic	21	21	21
251.8	Abnormality of secretion of glucagon or gastrin	3	endocrine/metabolic	0	0	0
252	Disorders of parathyroid gland	3	endocrine/metabolic	34	34	34
252.1	Hyperparathyroidism	3	endocrine/metabolic	19	19	19
252.2	Hypoparathyroidism	3	endocrine/metabolic	2	2	2
253	Disorders of the pituitary gland and its hypothalamic control	3	endocrine/metabolic	34	34	34
253.1	Pituitary hyperfunction	3	endocrine/metabolic	13	13	13
253.11	Acromegaly and gigantism	3	endocrine/metabolic	0	0	0
253.2	Pituitary hypofunction	3	endocrine/metabolic	4	4	4
253.3	Diabetes insipidus	3	endocrine/metabolic	1	1	1
253.4	Anterior pituitary disorders	3	endocrine/metabolic	3	3	3
253.5	Pituitary dwarfism	3	endocrine/metabolic	2	2	2
253.7	Other disorders of neurohypophysis	3	endocrine/metabolic	3	3	3
254	Diseases of thymus gland	3	endocrine/metabolic	0	0	0
255	Disorders of adrenal glands	3	endocrine/metabolic	44	44	44
255.1	Adrenal hyperfunction	3	endocrine/metabolic	5	5	5

255.11	Cushing's syndrome	3	endocrine/metabolic	0	0	0
255.12	Hyperaldosteronism	3	endocrine/metabolic	1	1	1
255.13	Medulloadrenal hyperfunction	3	endocrine/metabolic	0	0	0
255.2	Adrenal hypofunction	3	endocrine/metabolic	21	21	21
255.21	Glucocorticoid deficiency	3	endocrine/metabolic	21	21	21
255.22	Mineralocorticoid deficiency	3	endocrine/metabolic	0	0	0
255.3	Adrenogenital disorders	3	endocrine/metabolic	0	0	0
256	Ovarian dysfunction	3	endocrine/metabolic	80	80	80
256.1	Hyperestrogenism	3	endocrine/metabolic	0	0	0
256.4	Polycystic ovaries	3	endocrine/metabolic	70	70	70
257	Testicular dysfunction	3	endocrine/metabolic	86	86	86
257.1	Testicular hypofunction	3	endocrine/metabolic	81	81	81
258	Iatrogenic endocrine disorders	3	endocrine/metabolic	1	1	1
258.1	Postablative ovarian failure	3	endocrine/metabolic	5	5	5
259	Other endocrine disorders	3	endocrine/metabolic	48	48	48
259.1	Nonspecific abnormal results of other endocrine function study	3	endocrine/metabolic	1	1	1
259.2	Carcinoid syndrome	3	endocrine/metabolic	0	0	0
259.3	Delay in sexual development and puberty NEC	3	endocrine/metabolic	0	0	0
259.4	Precocious sexual development and puberty NEC	3	endocrine/metabolic	1	1	1
259.8	Polyglandular activity in multiple endocrine adenomatosis	3	endocrine/metabolic	1	1	1
260	Protein-calorie malnutrition	3	endocrine/metabolic	27	27	27
260.1	Cachexia	3	endocrine/metabolic	0	0	0
260.2	severe protein-calorie malnutrition	3	endocrine/metabolic	10	10	10
260.21	Kwashiorkor	3	endocrine/metabolic	0	0	0
260.22	Nutritional marasmus	3	endocrine/metabolic	0	0	0
260.3	Adult failure to thrive	3	endocrine/metabolic	4	4	4
260.6	Anorexia	3	endocrine/metabolic	0	0	0
260.7	Polyphagia	3	endocrine/metabolic	7	7	7
261	Vitamin deficiency	3	endocrine/metabolic	1192	1192	1192
261.1	Vitamin A deficiency	3	endocrine/metabolic	0	0	0
261.2	Vitamin B-complex deficiencies	3	endocrine/metabolic	112	112	112
261.3	Vitamin C deficiencies	3	endocrine/metabolic	0	0	0
261.4	Vitamin D deficiency	3	endocrine/metabolic	1135	1135	1135
261.41	Rickets or osteomalacia	3	endocrine/metabolic	0	0	0
262	Mineral deficiency NEC	3	endocrine/metabolic	2	2	2
263	Other nutritional deficiency	3	endocrine/metabolic	1	1	1
264	Lack of normal physiological development	3	endocrine/metabolic	2	2	2
264.1	Short stature	3	endocrine/metabolic	1	1	1
264.2	Failure to thrive (childhood)	3	endocrine/metabolic	1	1	1
264.3	Delayed milestones	3	endocrine/metabolic	0	0	0
264.9	Lack of normal physiological development, unspecified	3	endocrine/metabolic	0	0	0
269	Proteinuria	3	endocrine/metabolic	12	12	12
270	Disorders of protein plasma/amino-acid transport and metabolism	3	endocrine/metabolic	40	40	40
270.1	Disturbances of amino-acid transport	3	endocrine/metabolic	15	15	15
270.11	Disturbances of sulphur-bearing amino-acid metabolism	3	endocrine/metabolic	15	15	15
270.12	Phenylketonuria [PKU]	3	endocrine/metabolic	0	0	0
270.2	Disorders of amino-acid metabolism	3	endocrine/metabolic	2	2	2
270.21	Disorders of urea cycle metabolism	3	endocrine/metabolic	1	1	1
270.3	Disorders of plasma protein metabolism	3	endocrine/metabolic	21	21	21
270.31	Polyclonal hypergammaglobulinemia	3	endocrine/metabolic	0	0	0
270.32	Paraproteinemia	3	endocrine/metabolic	5	5	5
270.33	Amyloidosis	3	endocrine/metabolic	1	1	1
270.34	Alpha-1-antitrypsin deficiency	3	endocrine/metabolic	5	5	5
270.35	Macroglobulinemia	3	endocrine/metabolic	0	0	0
270.38	Other specified disorders of plasma protein metabolism	3	endocrine/metabolic	11	11	11
271	Disorders of carbohydrate transport and metabolism	3	endocrine/metabolic	8	8	8
271.3	Intestinal disaccharidase deficiencies and disaccharide malabsorption	3	endocrine/metabolic	6	6	6
271.9	Other disorders of carbohydrate transport and metabolism	3	endocrine/metabolic	2	2	2
272	Disorders of lipoid metabolism	3	endocrine/metabolic	1922	1922	1922
272.1	Hyperlipidemia	3	endocrine/metabolic	1793	1793	1793
272.11	Hypercholesterolemia	3	endocrine/metabolic	142	142	142
272.12	Hyperglyceridemia	3	endocrine/metabolic	117	117	117
272.13	Mixed hyperlipidemia	3	endocrine/metabolic	345	345	345
272.14	Hyperchylomicronemia	3	endocrine/metabolic	0	0	0
272.9	Unspecified disorder of lipoid metabolism	3	endocrine/metabolic	1	1	1
274	Gout and other crystal arthropathies	3	endocrine/metabolic	121	121	121
274.1	Gout	3	endocrine/metabolic	116	116	116

274.11	Gouty arthropathy	3	endocrine/metabolic	41	41	41
274.2	Crystal arthropathies	3	endocrine/metabolic	6	6	6
274.21	Chondrocalcinosis	3	endocrine/metabolic	5	5	5
275	Disorders of mineral metabolism	3	endocrine/metabolic	144	144	144
275.1	Disorders of iron metabolism	4	hematopoietic	35	35	35
275.11	Hereditary hemochromatosis	4	hematopoietic	7	7	7
275.2	Disorders of copper metabolism	3	endocrine/metabolic	1	1	1
275.3	Disorders of magnesium metabolism	3	endocrine/metabolic	19	19	19
275.5	Disorders of calcium/phosphorus metabolism	3	endocrine/metabolic	46	46	46
275.51	Hypocalcemia	3	endocrine/metabolic	19	19	19
275.53	Disorders of phosphorus metabolism	3	endocrine/metabolic	18	18	18
275.6	Hypercalcemia	3	endocrine/metabolic	38	38	38
276	Disorders of fluid, electrolyte, and acid-base balance	3	endocrine/metabolic	429	429	429
276.1	Electrolyte imbalance	3	endocrine/metabolic	388	388	388
276.11	Hyperosmolality and/or hypernatremia	3	endocrine/metabolic	0	0	0
276.12	Hyposmolality and/or hyponatremia	3	endocrine/metabolic	137	137	137
276.13	Hyperpotassemia	3	endocrine/metabolic	16	16	16
276.14	Hypopotassemia	3	endocrine/metabolic	254	254	254
276.4	Acid-base balance disorder	3	endocrine/metabolic	33	33	33
276.41	Acidosis	3	endocrine/metabolic	32	32	32
276.42	Alkalosis	3	endocrine/metabolic	0	0	0
276.5	Hypovolemia	3	endocrine/metabolic	64	64	64
276.6	Fluid overload	3	endocrine/metabolic	10	10	10
276.8	Polydipsia	3	endocrine/metabolic	3	3	3
277	Other disorders of metabolism	3	endocrine/metabolic	59	59	59
277.1	Disorders of porphyrin metabolism	3	endocrine/metabolic	1	1	1
277.2	Other disorders of purine and pyrimidine metabolism	3	endocrine/metabolic	0	0	0
277.4	Disorders of bilirubin excretion	3	endocrine/metabolic	18	18	18
277.5	Other disorders of lipoid metabolism	3	endocrine/metabolic	39	39	39
277.51	Lipoprotein disorders	3	endocrine/metabolic	34	34	34
277.6	Other deficiencies of circulating enzymes	3	endocrine/metabolic	0	0	0
277.7	Dysmetabolic syndrome X	3	endocrine/metabolic	30	30	30
277.8	Carnitine deficiencies	3	endocrine/metabolic	0	0	0
278	Overweight, obesity and other hyperalimentation	3	endocrine/metabolic	1325	1325	1325
278.1	Obesity	3	endocrine/metabolic	826	826	826
278.11	Morbid obesity	3	endocrine/metabolic	490	490	490
278.3	Localized adiposity	3	endocrine/metabolic	4	4	4
278.4	Abnormal weight gain	3	endocrine/metabolic	122	122	122
279	Disorders involving the immune mechanism	3	endocrine/metabolic	31	31	31
279.1	Immunity deficiency	3	endocrine/metabolic	15	15	15
279.11	Deficiency of humoral immunity	3	endocrine/metabolic	9	9	9
279.2	Autoimmune disease NEC	3	endocrine/metabolic	4	4	4
279.7	Other immunological findings	3	endocrine/metabolic	24	24	24
279.8	Other specified disorders involving the immune mechanism	3	endocrine/metabolic	2	2	2
280	Iron deficiency anemias	4	hematopoietic	124	124	124
280.1	Iron deficiency anemias unspecified or not due to blood loss	4	hematopoietic	107	107	107
280.2	Iron deficiency anemia secondary to blood loss (chronic)	4	hematopoietic	0	0	0
281	Other deficiency anemia	4	hematopoietic	28	28	28
281.1	Megaloblastic anemia	4	hematopoietic	9	9	9
281.11	Pernicious anemia	4	hematopoietic	0	0	0
281.12	Other vitamin B12 deficiency anemia	4	hematopoietic	6	6	6
281.13	Folate-deficiency anemia	4	hematopoietic	0	0	0
281.9	Deficiency anemias	4	hematopoietic	12	12	12
282	Hereditary hemolytic anemias	4	hematopoietic	24	24	24
282.5	Sickle cell anemia	4	hematopoietic	2	2	2
282.8	Other hemoglobinopathies	4	hematopoietic	21	21	21
282.9	Other hereditary hemolytic anemias	4	hematopoietic	1	1	1
283	Acquired hemolytic anemias	4	hematopoietic	2	2	2
283.1	Autoimmune hemolytic anemias	4	hematopoietic	0	0	0
283.2	Non-autoimmune hemolytic anemias	4	hematopoietic	0	0	0
283.21	Hemolytic-uremic syndrome	4	hematopoietic	0	0	0
284	Aplastic anemia	4	hematopoietic	17	17	17
284.1	Pancytopenia	4	hematopoietic	15	15	15
284.2	Constitutional aplastic anemia	4	hematopoietic	1	1	1
285	Other anemias	4	hematopoietic	311	311	311
285.1	Acute posthemorrhagic anemia	4	hematopoietic	22	22	22
285.2	Anemia of chronic disease	4	hematopoietic	9	9	9

285.21	Anemia in chronic kidney disease	4	hematopoietic	3	3	3
285.22	Anemia in neoplastic disease	4	hematopoietic	2	2	2
285.3	Sideroblastic anemia	4	hematopoietic	0	0	0
285.8	Hemoglobinuria	4	hematopoietic	0	0	0
286	Coagulation defects	4	hematopoietic	78	78	78
286.1	Congenital coagulation defects	4	hematopoietic	6	6	6
286.11	Von willebrand's disease	4	hematopoietic	3	3	3
286.12	Congenital deficiency of other clotting factors (including factor VII)	4	hematopoietic	3	3	3
286.13	Congenital factor VIII disorder	4	hematopoietic	0	0	0
286.2	Encounter for long-term (current) use of anticoagulants	4	hematopoietic	127	127	127
286.3	Coagulation defects complicating pregnancy or postpartum	4	hematopoietic	6	6	6
286.4	Acquired coagulation factor deficiency	4	hematopoietic	2	2	2
286.5	Hemorrhagic disorder due to intrinsic circulating anticoagulants	4	hematopoietic	3	3	3
286.6	Defibrination syndrome	4	hematopoietic	0	0	0
286.7	Other and unspecified coagulation defects	4	hematopoietic	20	20	20
286.8	Hypercoagulable state	4	hematopoietic	30	30	30
286.81	Primary hypercoagulable state	4	hematopoietic	29	29	29
286.9	Abnormal coagulation profile	4	hematopoietic	15	15	15
287	Purpura and other hemorrhagic conditions	4	hematopoietic	102	102	102
287.1	Spontaneous ecchymoses	4	hematopoietic	8	8	8
287.2	Allergic purpura	4	hematopoietic	0	0	0
287.3	Thrombocytopenia	4	hematopoietic	81	81	81
287.31	Primary thrombocytopenia	4	hematopoietic	5	5	5
287.32	Secondary thrombocytopenia	4	hematopoietic	3	3	3
287.4	Qualitative platelet defects	4	hematopoietic	4	4	4
288	Diseases of white blood cells	4	hematopoietic	181	181	181
288.1	Decreased white blood cell count	4	hematopoietic	104	104	104
288.11	Neutropenia	4	hematopoietic	22	22	22
288.2	Elevated white blood cell count	4	hematopoietic	148	148	148
288.3	Eosinophilia	4	hematopoietic	3	3	3
289	Other diseases of blood and blood-forming organs	4	hematopoietic	101	101	101
289.1	Myelofibrosis	4	hematopoietic	0	0	0
289.3	Personal history of diseases of blood and blood-forming organs	4	hematopoietic	24	24	24
289.4	Lymphadenitis	4	hematopoietic	118	118	118
289.5	Diseases of spleen	4	hematopoietic	3	3	3
289.8	Polycythemia vera, secondary	4	hematopoietic	0	0	0
289.9	Abnormality of red blood cells	4	hematopoietic	16	16	16
290	Delirium dementia and amnestic and other cognitive disorders	5	mental disorders	19	19	19
290.1	Dementias	5	mental disorders	15	15	15
290.11	Alzheimer's disease	5	mental disorders	5	5	5
290.12	Dementia with cerebral degenerations	5	mental disorders	1	1	1
290.13	Senile dementia	5	mental disorders	0	0	0
290.16	Vascular dementia	5	mental disorders	3	3	3
290.2	Delirium due to conditions classified elsewhere	5	mental disorders	0	0	0
290.3	Other persistent mental disorders due to conditions classified elsewhere	5	mental disorders	6	6	6
291	Other specified nonpsychotic and/or transient mental disorders	5	mental disorders	25	25	25
291.1	Transient mental disorders due to conditions classified elsewhere	5	mental disorders	1	1	1
291.4	Specific nonpsychotic mental disorders due to brain damage	5	mental disorders	16	16	16
291.8	Alteration of consciousness	5	mental disorders	6	6	6
292	Neurological disorders	5	mental disorders	166	166	166
292.1	Aphasia/speech disturbance	5	mental disorders	30	30	30
292.11	Aphasia	5	mental disorders	6	6	6
292.12	Symbolic dysfunction	5	mental disorders	1	1	1
292.2	Mild cognitive impairment	5	mental disorders	11	11	11
292.3	Memory loss	5	mental disorders	76	76	76
292.4	Altered mental status	5	mental disorders	38	38	38
292.5	Transient alteration of awareness	5	mental disorders	2	2	2
292.6	Hallucinations	5	mental disorders	3	3	3
293	Symptoms involving head and neck	5	mental disorders	303	303	303
293.1	Swelling, mass, or lump in head and neck [Space-occupying lesion, intracranial NOS]	5	mental disorders	75	75	75
295	Schizophrenia and other psychotic disorders	5	mental disorders	15	15	15
295.1	Schizophrenia	5	mental disorders	4	4	4
295.2	Paranoid disorders	5	mental disorders	1	1	1
295.3	Psychosis	5	mental disorders	11	11	11
296	Mood disorders	5	mental disorders	643	643	643
296.1	Bipolar	5	mental disorders	57	57	57
296.2	Depression	5	mental disorders	567	567	567

296.22	Major depressive disorder	5	mental disorders	252	252	252
297	Suicidal ideation or attempt	5	mental disorders	18	18	18
297.1	Suicidal ideation	5	mental disorders	7	7	7
297.2	Suicide or self-inflicted injury	5	mental disorders	8	8	8
300	Anxiety, phobic and dissociative disorders	5	mental disorders	951	951	951
300.1	Anxiety disorder	5	mental disorders	380	380	380
300.11	Generalized anxiety disorder	5	mental disorders	109	109	109
300.12	Agorophobia, social phobia, and panic disorder	5	mental disorders	70	70	70
300.13	Phobia	5	mental disorders	29	29	29
300.2	Generalized anxiety & phobic disorders	5	mental disorders	0	0	0
300.3	Obsessive-compulsive disorders	5	mental disorders	12	12	12
300.4	Dysthymic disorder	5	mental disorders	32	32	32
300.8	Acute reaction to stress	5	mental disorders	21	21	21
300.9	Posttraumatic stress disorder	5	mental disorders	55	55	55
301	Personality disorders	5	mental disorders	3	3	3
301.1	Schizoid personality disorder	5	mental disorders	0	0	0
301.2	Antisocial/borderline personality disorder	5	mental disorders	2	2	2
302	Sexual and gender identity disorders	5	mental disorders	31	31	31
302.1	Decreased libido	5	mental disorders	26	26	26
303	Psychogenic and somatoform disorders	5	mental disorders	4	4	4
303.1	Dissociative disorder	5	mental disorders	1	1	1
303.3	Psychogenic disorder	5	mental disorders	2	2	2
303.31	Gastrointestinal malfunction arising from mental factors	5	mental disorders	0	0	0
303.4	Somatoform disorder	5	mental disorders	2	2	2
304	Adjustment reaction	5	mental disorders	119	119	119
305.2	Eating disorder	5	mental disorders	10	10	10
305.21	Anorexia nervosa	5	mental disorders	1	1	1
306	Other mental disorder	5	mental disorders	22	22	22
306.1	Mental disorders durring/after pregnancy	5	mental disorders	10	10	10
306.9	Tension headache	5	mental disorders	3	3	3
312	Conduct disorders	5	mental disorders	4	4	4
312.3	Impulse control disorder	5	mental disorders	2	2	2
313	Pervasive developmental disorders	5	mental disorders	68	68	68
313.1	Attention deficit hyperactivity disorder	5	mental disorders	63	63	63
313.2	Tics and stuttering	5	mental disorders	2	2	2
313.3	Autism	5	mental disorders	1	1	1
315	Develomental delays and disorders	5	mental disorders	9	9	9
315.1	Learning disorder	5	mental disorders	0	0	0
315.2	Speech and language disorder	5	mental disorders	4	4	4
315.3	Mental retardation	5	mental disorders	1	1	1
316	Substance addiction and disorders	5	mental disorders	118	118	118
316.1	Polyneuropathy due to drugs	5	mental disorders	4	4	4
317	Alcohol-related disorders	5	mental disorders	43	43	43
317.1	Alcoholism	5	mental disorders	23	23	23
317.11	Alcoholic liver damage	5	mental disorders	11	11	11
318	Tobacco use disorder	5	mental disorders	197	197	197
320	Meningitis	6	neurological	4	4	4
323	Encephalitis	6	neurological	4	4	4
323.2	Acute (transverse) myelitis	6	neurological	0	0	0
323.8	Encephalitis, non-infectious	6	neurological	4	4	4
324	Other CNS infection and poliomyelitis	6	neurological	9	9	9
324.1	Jakob-Creutzfeldt disease	6	neurological	0	0	0
325	Phlebitis and thrombophlebitis of intracranial venous sinuses	6	neurological	1	1	1
327	Sleep disorders	6	neurological	626	626	626
327.1	Hypersomnia	6	neurological	17	17	17
327.3	Sleep apnea	6	neurological	467	467	467
327.31	Central/nonobstroctive sleep apnea	6	neurological	59	59	59
327.32	Obstructive sleep apnea	6	neurological	346	346	346
327.4	Insomnia	6	neurological	496	496	496
327.41	Organic or persistent insomnia	6	neurological	150	150	150
327.5	Parasomnia	6	neurological	4	4	4
327.6	Circadian rhythm sleep disorder	6	neurological	15	15	15
327.7	Sleep related movement disorders	6	neurological	70	70	70
327.71	Restless legs syndrome	6	neurological	58	58	58
327.72	Sleep related leg cramps	6	neurological	12	12	12
331	Other cerebral degenerations	6	neurological	12	12	12
331.1	Hydrocephalus	6	neurological	1	1	1

331.9	Cerebral degeneration, unspecified	6	neurological	7	7	7
332	Parkinson's disease	6	neurological	8	8	8
333	Extrapyramidal disease and abnormal movement disorders	6	neurological	42	42	42
333.1	Essential tremor	6	neurological	36	36	36
333.2	Myoclonus	6	neurological	1	1	1
333.3	Tics and choreas	6	neurological	0	0	0
333.4	Torsion dystonia	6	neurological	2	2	2
333.8	Other degenerative diseases of the basal ganglia	6	neurological	0	0	0
334	Degenerative disease of the spinal cord	6	neurological	11	11	11
334.1	Spinocerebellar disease	6	neurological	0	0	0
334.2	Anterior horn cell disease	6	neurological	1	1	1
334.21	Amyotrophic Lateral Sclerosis	6	neurological	1	1	1
335	Multiple sclerosis	6	neurological	38	38	38
337	Disorders of the autonomic nervous system	6	neurological	7	7	7
337.1	Peripheral autonomic neuropathy	6	neurological	3	3	3
338	Pain	6	neurological	812	812	812
338.1	Acute pain	6	neurological	89	89	89
338.2	Chronic pain	6	neurological	745	745	745
339	Other headache syndromes	6	neurological	86	86	86
340	Migraine	6	neurological	457	457	457
340.1	Migrain with aura	6	neurological	46	46	46
341	Other demyelinating diseases of central nervous system	6	neurological	4	4	4
342	Hemiplegia	6	neurological	2	2	2
343	Infantile cerebral palsy	6	neurological	2	2	2
344	Other paralytic syndromes	6	neurological	7	7	7
345	Epilepsy, recurrent seizures, convulsions	6	neurological	90	90	90
345.1	Epilepsy	6	neurological	21	21	21
345.11	Generalized convulsive epilepsy	6	neurological	1	1	1
345.12	Partial epilepsy	6	neurological	16	16	16
345.3	Convulsions	6	neurological	58	58	58
346	Abnormal findings on study of brain and/or nervous system	6	neurological	4	4	4
346.1	Nonspecific abnormal findings on radiological and other examination of skull and head	6	neurological	3	3	3
346.2	Nonspecific abnormal results of function study of brain and central nervous system	6	neurological	3	3	3
346.3	Nonspecific abnormal findings in cerebrospinal fluid	6	neurological	0	0	0
347	Cataplexy and narcolepsy	6	neurological	5	5	5
348	Other conditions of brain	6	neurological	34	34	34
348.2	Cerebral edema and compression of brain	6	neurological	14	14	14
348.4	Cerebral cysts	6	neurological	0	0	0
348.7	Coma	6	neurological	1	1	1
348.8	Encephalopathy, not elsewhere classified	6	neurological	16	16	16
348.9	Other conditions of brain, NOS	6	neurological	16	16	16
349	Other and unspecified disorders of the nervous system	6	neurological	3	3	3
350	Abnormal movement	6	neurological	65	65	65
350.1	Abnormal involuntary movements	6	neurological	1	1	1
350.2	Abnormality of gait	6	neurological	39	39	39
350.3	Lack of coordination	6	neurological	16	16	16
350.5	Abnormal reflex	6	neurological	4	4	4
350.6	Disturbances of sensation of smell and taste	6	neurological	13	13	13
351	Other peripheral nerve disorders	6	neurological	198	198	198
352	Disorders of other cranial nerves	6	neurological	32	32	32
352.1	Trigeminal nerve disorders [CN5]	6	neurological	12	12	12
352.2	Facial nerve disorders [CN7]	6	neurological	19	19	19
353	Nerve root and plexus disorders	6	neurological	3	3	3
353.1	Nerve plexus lesions	6	neurological	1	1	1
353.2	Nerve root lesions	6	neurological	0	0	0
355	Complex regional/central pain syndrome	6	neurological	10	10	10
355.1	Chronic pain syndrome	6	neurological	42	42	42
356	Hereditary and idiopathic peripheral neuropathy	6	neurological	54	54	54
357	Inflammatory and toxic neuropathy	6	neurological	31	31	31
358	Myoneural disorders	6	neurological	3	3	3
358.1	Myasthenia gravis	6	neurological	0	0	0
359	Muscular dystrophies and other myopathies	6	neurological	7	7	7
359.1	Muscular dystrophies	6	neurological	1	1	1
359.2	Myopathy	6	neurological	6	6	6
360	Disorders of the globe	7	sense organs	0	0	0
360.2	Progressive myopia	7	sense organs	0	0	0
360.3	Hypotony of eye	7	sense organs	0	0	0

361	Retinal detachments and defects	7	sense organs	13	13	13
361.1	Retinal detachment with retinal defect	7	sense organs	6	6	6
361.2	Retinoschisis and retinal cysts	7	sense organs	0	0	0
362	Other retinal disorders	7	sense organs	47	47	47
362.1	Retinopathy of prematurity	7	sense organs	0	0	0
362.2	Degeneration of macula and posterior pole of retina	7	sense organs	25	25	25
362.21	Macular degeneration, dry	7	sense organs	0	0	0
362.22	Macular degeneration, wet	7	sense organs	1	1	1
362.23	Cystoid macular degeneration of retina	7	sense organs	2	2	2
362.26	Macular puckering of retina	7	sense organs	1	1	1
362.27	Drusen (degenerative) of retina	7	sense organs	1	1	1
362.29	Macular degeneration (senile) of retina NOS	7	sense organs	0	0	0
362.3	Other nondiabetic retinopathy	7	sense organs	0	0	0
362.31	Separation of retinal layers	7	sense organs	0	0	0
362.4	Retinal vascular changes and abnomalities	7	sense organs	13	13	13
362.5	Toxic maculopathy of retina	7	sense organs	0	0	0
362.6	Peripheral retinal degenerations	7	sense organs	0	0	0
362.7	Hereditary retinal dystrophies	7	sense organs	3	3	3
362.8	Retinal hemorrhage/ischemia	7	sense organs	3	3	3
362.9	Retinal edema	7	sense organs	0	0	0
363	Chorioretinal inflammations, scars, and other disorders of choroid	7	sense organs	0	0	0
363.3	Chorioretinal scars	7	sense organs	0	0	0
363.4	Choroidal degenerations	7	sense organs	0	0	0
364	Corneal opacity and other disorders of cornea	7	sense organs	10	10	10
364.1	Corneal opacity	7	sense organs	0	0	0
364.2	Corneal edema	7	sense organs	0	0	0
364.4	Corneal degenerations	7	sense organs	3	3	3
364.41	Keratoconus	7	sense organs	2	2	2
364.5	Corneal dystrophy	7	sense organs	1	1	1
364.51	Fuchs' dystrophy	7	sense organs	0	0	0
364.9	Cornea replaced by transplant	7	sense organs	3	3	3
365	Glaucoma	7	sense organs	62	62	62
365.1	Open-angle glaucoma	7	sense organs	8	8	8
365.11	Primary open angle glaucoma	7	sense organs	3	3	3
365.2	Primary angle-closure glaucoma	7	sense organs	4	4	4
365.5	Pseudoexfoliation glaucoma	7	sense organs	0	0	0
366	Cataract	7	sense organs	154	154	154
366.1	Nonsenile Cataract	7	sense organs	0	0	0
366.2	Senile cataract	7	sense organs	58	58	58
366.3	Traumatic cataract	7	sense organs	0	0	0
367	Disorders of refraction and accommodation; blindness and low vision	7	sense organs	26	26	26
367.1	Myopia	7	sense organs	4	4	4
367.2	Astigmatism	7	sense organs	5	5	5
367.8	Hypermetropia	7	sense organs	0	0	0
367.9	Blindness and low vision	7	sense organs	13	13	13
368	Visual disturbances	7	sense organs	98	98	98
368.1	Amblyopia	7	sense organs	0	0	0
368.2	Diplopia and disorders of binocular vision	7	sense organs	10	10	10
368.3	Anisometropia	7	sense organs	0	0	0
368.4	Visual field defects	7	sense organs	18	18	18
368.5	Color vision deficiencies	7	sense organs	0	0	0
368.7	Disorders of accommodation	7	sense organs	0	0	0
368.9	Subjective visual disturbances	7	sense organs	13	13	13
368.91	Psychophysical visual disturbances	7	sense organs	1	1	1
369	Infection of the eye	7	sense organs	167	167	167
369.2	Eye infection, viral	7	sense organs	1	1	1
369.5	Conjunctivitis, infectious	7	sense organs	163	163	163
370	Keratitis	7	sense organs	3	3	3
370.1	Corneal ulcer	7	sense organs	1	1	1
370.2	Superficial keratitis	7	sense organs	2	2	2
370.3	Keratoconjunctivitis	7	sense organs	0	0	0
370.31	Keratoconjunctivitis sicca	7	sense organs	0	0	0
371	Inflammation of the eye	7	sense organs	96	96	96
371.1	Uveitis, noninfectious or NOS	7	sense organs	7	7	7
371.2	Conjunctivitis, noninfectious	7	sense organs	12	12	12
371.21	Allergic conjunctivitis	7	sense organs	11	11	11
371.3	Inflammation of eyelids	7	sense organs	53	53	53

371.33	Noninfectious dermatoses of eyelid	7	sense organs	3	3	3
371.9	Chronic inflammatory disorders of orbit	7	sense organs	0	0	0
372	Disorders of conjunctiva	7	sense organs	22	22	22
374	Other disorders of eyelids	7	sense organs	48	48	48
374.1	Ectropion or entropion	7	sense organs	3	3	3
374.2	Lagophthalmos	7	sense organs	0	0	0
374.3	Ptosis of eyelid	7	sense organs	19	19	19
374.6	Dermatochalasis	7	sense organs	10	10	10
375	Disorders of lacrimal system	7	sense organs	4	4	4
375.1	Dry eyes	7	sense organs	25	25	25
375.2	Epiphora	7	sense organs	1	1	1
376	Disorders of the orbit	7	sense organs	2	2	2
377	Disorders of optic nerve and visual pathways	7	sense organs	28	28	28
377.1	Optic atrophy	7	sense organs	2	2	2
377.3	Optic neuritis/neuropathy	7	sense organs	20	20	20
378	Strabismus and other disorders of binocular eye movements	7	sense organs	14	14	14
378.1	Strabismus (not specified as paralytic)	7	sense organs	3	3	3
378.2	Nystagmus and other irregular eye movements	7	sense organs	1	1	1
378.5	Paralytic strabismus	7	sense organs	4	4	4
379	Other disorders of eye	7	sense organs	35	35	35
379.1	Scleritis and episcleritis	7	sense organs	0	0	0
379.2	Disorders of vitreous body	7	sense organs	17	17	17
379.3	Aphakia and other disorders of lens	7	sense organs	1	1	1
379.4	Anomalies of pupillary function	7	sense organs	5	5	5
379.5	Disorders of iris and ciliary body	7	sense organs	1	1	1
379.51	Pigmentary iris degeneration	7	sense organs	0	0	0
379.9	Pain, swelling or discharge of eye	7	sense organs	29	29	29
380	Disorders of external ear	7	sense organs	3	3	3
380.1	Otitis externa	7	sense organs	60	60	60
380.4	Impacted cerumen	7	sense organs	135	135	135
381	Otitis media and Eustachian tube disorders	7	sense organs	345	345	345
381.1	Otitis media	7	sense organs	238	238	238
381.11	Suppurative and unspecified otitis media	7	sense organs	168	168	168
381.2	Eustachian tube disorders	7	sense organs	75	75	75
381.3	Mastoiditis & related conditions	7	sense organs	9	9	9
381.9	Otorrhea	7	sense organs	3	3	3
382	Otalgia	7	sense organs	173	173	173
383	Otosclerosis	7	sense organs	1	1	1
384	Other disorders of tympanic membrane	7	sense organs	12	12	12
384.1	Myringitis	7	sense organs	4	4	4
384.4	Perforation of tympanic membrane	7	sense organs	7	7	7
385	Other disorders of middle ear and mastoid	7	sense organs	6	6	6
385.3	Cholesteatoma	7	sense organs	4	4	4
385.5	Tympanosclerosis and middle ear disease related to otitis media	7	sense organs	1	1	1
386	Vertiginous syndromes and other disorders of vestibular system	7	sense organs	76	76	76
386.1	Meniere's disease	7	sense organs	0	0	0
386.2	Peripheral or central vertigo	7	sense organs	60	60	60
386.21	Central origin vertigo	7	sense organs	0	0	0
386.3	Labyrinthitis	7	sense organs	2	2	2
386.9	Dizziness and giddiness (Light-headedness and vertigo)	7	sense organs	401	401	401
388	Other disorders of ear	7	sense organs	35	35	35
389	Hearing loss	7	sense organs	168	168	168
389.1	Sensorineural hearing loss	7	sense organs	1	1	1
389.2	Conductive hearing loss	7	sense organs	1	1	1
389.3	Degenerative and vascular disorders of ear	7	sense organs	5	5	5
389.4	Tinnitus	7	sense organs	36	36	36
389.5	Disorders of acoustic nerve	7	sense organs	1	1	1
394	Rheumatic disease of the heart valves	8	circulatory system	66	66	66
394.1	Mitral valve stenosis and aortic valve stenosis	8	circulatory system	5	5	5
394.2	Mitral valve disease	8	circulatory system	12	12	12
394.3	Aortic valve disease	8	circulatory system	1	1	1
394.4	Acute rheumatic heart disease	8	circulatory system	1	1	1
394.7	Disease of tricuspid valve	8	circulatory system	0	0	0
395	Heart valve disorders	8	circulatory system	331	331	331
395.1	Nonrheumatic mitral valve disorders	8	circulatory system	47	47	47
395.2	Nonrheumatic aortic valve disorders	8	circulatory system	93	93	93
395.3	Nonrheumatic tricuspid valve disorders	8	circulatory system	43	43	43

395.4	Nonrheumatic pulmonary valve disorders	8	circulatory system	12	12	12
395.6	Heart valve replaced	8	circulatory system	22	22	22
396	Abnormal heart sounds	8	circulatory system	101	101	101
401	Hypertension	8	circulatory system	1242	1242	1242
401.1	Essential hypertension	8	circulatory system	1229	1229	1229
401.2	Hypertensive heart and/or renal disease	8	circulatory system	9	9	9
401.21	Hypertensive heart disease	8	circulatory system	2	2	2
401.22	Hypertensive chronic kidney disease	8	circulatory system	5	5	5
401.3	Other hypertensive complications	8	circulatory system	43	43	43
411	Ischemic Heart Disease	8	circulatory system	254	254	254
411.1	Unstable angina (intermediate coronary syndrome)	8	circulatory system	14	14	14
411.2	Myocardial infarction	8	circulatory system	97	97	97
411.3	Angina pectoris	8	circulatory system	36	36	36
411.4	Coronary atherosclerosis	8	circulatory system	158	158	158
411.41	Aneurysm and dissection of heart	8	circulatory system	1	1	1
411.8	Other chronic ischemic heart disease, unspecified	8	circulatory system	115	115	115
411.9	Other acute and subacute forms of ischemic heart disease	8	circulatory system	0	0	0
414	Other forms of chronic heart disease	8	circulatory system	52	52	52
414.2	ASCVD	8	circulatory system	2	2	2
415	Pulmonary heart disease	8	circulatory system	97	97	97
415.1	Acute pulmonary heart disease	8	circulatory system	37	37	37
415.11	Pulmonary embolism and infarction, acute	8	circulatory system	37	37	37
415.2	Chronic pulmonary heart disease	8	circulatory system	48	48	48
415.21	Primary pulmonary hypertension	8	circulatory system	0	0	0
416	Cardiomegaly	8	circulatory system	54	54	54
418	Nonspecific chest pain	8	circulatory system	764	764	764
418.1	Precordial pain	8	circulatory system	26	26	26
420	Carditis	8	circulatory system	33	33	33
420.1	Myocarditis	8	circulatory system	1	1	1
420.2	Pericarditis	8	circulatory system	23	23	23
420.21	Acute pericarditis	8	circulatory system	2	2	2
420.22	Chronic pericarditis	8	circulatory system	3	3	3
420.3	Endocarditis	8	circulatory system	8	8	8
425	Cardiomyopathy	8	circulatory system	27	27	27
425.1	Primary/intrinsic cardiomyopathies	8	circulatory system	26	26	26
425.11	Hypertrophic obstructive cardiomyopathy	8	circulatory system	1	1	1
425.12	Other hypertrophic cardiomyopathy	8	circulatory system	4	4	4
425.2	Secondary/extrinsic cardiomyopathies	8	circulatory system	3	3	3
425.8	Other cardiomyopathy	8	circulatory system	2	2	2
426	Cardiac conduction disorders	8	circulatory system	241	241	241
426.2	Atrioventricular [AV] block	8	circulatory system	21	21	21
426.21	First degree AV block	8	circulatory system	9	9	9
426.22	Mobitz II AV block	8	circulatory system	1	1	1
426.23	Second degree AV block	8	circulatory system	2	2	2
426.24	Atrioventricular block, complete	8	circulatory system	0	0	0
426.25	Other heart block	8	circulatory system	3	3	3
426.3	Bundle branch block	8	circulatory system	53	53	53
426.31	Right bundle branch block	8	circulatory system	24	24	24
426.32	Left bundle branch block	8	circulatory system	23	23	23
426.4	Anomalous atrioventricular excitation	8	circulatory system	5	5	5
426.7	Abnormal electrocardiogram [ECG] [EKG]	8	circulatory system	117	117	117
426.8	Other cardiac conduction disorders	8	circulatory system	10	10	10
426.9	Cardiac pacemaker/device in situ	8	circulatory system	45	45	45
426.91	Cardiac pacemaker in situ	8	circulatory system	41	41	41
426.92	Cardiac defibrillator in situ	8	circulatory system	7	7	7
427	Cardiac dysrhythmias	8	circulatory system	655	655	655
427.1	Paroxysmal tachycardia, unspecified	8	circulatory system	31	31	31
427.11	Paroxysmal supraventricular tachycardia	8	circulatory system	0	0	0
427.12	Paroxysmal ventricular tachycardia	8	circulatory system	24	24	24
427.2	Atrial fibrillation and flutter	8	circulatory system	153	153	153
427.21	Atrial fibrillation	8	circulatory system	146	146	146
427.22	Atrial flutter	8	circulatory system	24	24	24
427.3	Other specified cardiac dysrhythmias	8	circulatory system	125	125	125
427.4	Cardiac arrest and ventricular fibrillation	8	circulatory system	9	9	9
427.41	Ventricular fibrillation and flutter	8	circulatory system	3	3	3
427.42	Cardiac arrest	8	circulatory system	6	6	6
427.5	Arrhythmia (cardiac) NOS	8	circulatory system	76	76	76

427.6	Premature beats	8	circulatory system	176	176	176
427.61	Supraventricular premature beats	8	circulatory system	76	76	76
427.7	Tachycardia NOS	8	circulatory system	0	0	0
427.8	Sinoatrial node dysfunction (Bradycardia)	8	circulatory system	27	27	27
427.9	Palpitations	8	circulatory system	279	279	279
428	Congestive heart failure; nonhypertensive	8	circulatory system	58	58	58
428.1	Congestive heart failure (CHF) NOS	8	circulatory system	11	11	11
428.2	Heart failure NOS	8	circulatory system	4	4	4
428.3	Heart failure with reduced EF [Systolic or combined heart failure]	8	circulatory system	13	13	13
428.4	Heart failure with preserved EF [Diastolic heart failure]	8	circulatory system	10	10	10
429	Ill-defined descriptions and complications of heart disease	8	circulatory system	99	99	99
429.1	Heart transplant/surgery	8	circulatory system	6	6	6
429.2	Abnormal function study of cardiovascular system	8	circulatory system	48	48	48
429.3	Symptoms involving cardiovascular system	8	circulatory system	48	48	48
429.9	Cardiac complications, not elsewhere classified	8	circulatory system	1	1	1
430	Intracranial hemorrhage	8	circulatory system	24	24	24
430.1	Subarachnoid hemorrhage	8	circulatory system	6	6	6
430.2	Intracerebral hemorrhage	8	circulatory system	8	8	8
430.3	Subdural hemorrhage	8	circulatory system	8	8	8
433	Cerebrovascular disease	8	circulatory system	218	218	218
433.1	Occlusion and stenosis of precerebral arteries	8	circulatory system	86	86	86
433.11	Occlusion of cerebral arteries, with cerebral infarction	8	circulatory system	6	6	6
433.12	Cerebral atherosclerosis	8	circulatory system	0	0	0
433.2	Occlusion of cerebral arteries	8	circulatory system	56	56	56
433.21	Cerebral artery occlusion, with cerebral infarction	8	circulatory system	55	55	55
433.3	Cerebral ischemia	8	circulatory system	97	97	97
433.31	Transient cerebral ischemia	8	circulatory system	95	95	95
433.32	Moyamoya disease	8	circulatory system	0	0	0
433.5	Cerebral aneurysm	8	circulatory system	11	11	11
433.6	Acute, but ill-defined cerebrovascular disease	8	circulatory system	3	3	3
433.8	Late effects of cerebrovascular disease	8	circulatory system	25	25	25
440	Atherosclerosis	8	circulatory system	76	76	76
440.1	Atherosclerosis of renal artery	8	circulatory system	2	2	2
440.2	Atherosclerosis of the extremities	8	circulatory system	13	13	13
440.21	Atherosclerosis of native arteries of the extremities with ulceration or gangrene	8	circulatory system	0	0	0
440.22	Atherosclerosis of native arteries of the extremities with intermittent claudication	8	circulatory system	4	4	4
440.9	Atherosclerosis of aorta	8	circulatory system	1	1	1
441	Vascular insufficiency of intestine	8	circulatory system	6	6	6
441.1	Acute vascular insufficiency of intestine	8	circulatory system	1	1	1
441.2	Chronic vascular insufficiency of intestine	8	circulatory system	0	0	0
442	Other aneurysm	8	circulatory system	47	47	47
442.1	Aortic aneurysm	8	circulatory system	41	41	41
442.11	Abdominal aortic aneurysm	8	circulatory system	22	22	22
442.2	Aneurysm of iliac artery	8	circulatory system	2	2	2
442.3	Aneurysm of artery of lower extremity	8	circulatory system	1	1	1
442.4	Arterial dissection	8	circulatory system	1	1	1
442.8	Aneurysm of other specified artery	8	circulatory system	0	0	0
443	Peripheral vascular disease	8	circulatory system	74	74	74
443.1	Raynaud's syndrome	8	circulatory system	0	0	0
443.7	Peripheral angiopathy in diseases classified elsewhere	8	circulatory system	6	6	6
443.8	Other specified peripheral vascular diseases	8	circulatory system	0	0	0
443.9	Peripheral vascular disease, unspecified	8	circulatory system	35	35	35
444	Arterial embolism and thrombosis	8	circulatory system	7	7	7
444.1	Arterial embolism and thrombosis of lower extremity artery	8	circulatory system	2	2	2
444.2	Embolism and thrombosis of abdominal aorta	8	circulatory system	0	0	0
444.5	Atheroembolism	8	circulatory system	0	0	0
446	Polyarteritis nodosa and allied conditions	8	circulatory system	10	10	10
446.1	Thromboangiitis obliterans	8	circulatory system	0	0	0
446.2	Acute febrile mucocutaneous lymph node syndrome (Kawasaki disease)	8	circulatory system	0	0	0
446.3	Hypersensitivity angiitis	8	circulatory system	0	0	0
446.4	Wegener's granulomatosis	8	circulatory system	1	1	1
446.5	Giant cell arteritis	8	circulatory system	3	3	3
446.6	Polyarteritis nodosa	8	circulatory system	0	0	0
446.7	Takayasu's disease	8	circulatory system	0	0	0
446.8	Thrombotic microangiopathy	8	circulatory system	0	0	0
446.9	Arteritis NOS	8	circulatory system	3	3	3
447	Other disorders of arteries and arterioles	8	circulatory system	54	54	54

447.1	Stricture of artery	8	circulatory system	6	6	6
447.7	Aortic ectasia	8	circulatory system	24	24	24
448	Disease of capillaries	8	circulatory system	1	1	1
450	Noninfectious disorders of lymphatic channels	8	circulatory system	22	22	22
451	Phlebitis and thrombophlebitis	8	circulatory system	23	23	23
451.2	Phlebitis and thrombophlebitis of lower extremities	8	circulatory system	8	8	8
452	Other venous embolism and thrombosis	8	circulatory system	130	130	130
452.1	Iatrogenic pulmonary embolism and infarction	8	circulatory system	5	5	5
452.2	Deep vein thrombosis [DVT]	8	circulatory system	72	72	72
452.8	Postphlebitic syndrome	8	circulatory system	2	2	2
453	Chronic venous hypertension	8	circulatory system	3	3	3
454	Varicose veins	8	circulatory system	57	57	57
454.1	Varicose veins of lower extremity	8	circulatory system	50	50	50
454.11	Varicose veins of lower extremity, symptomtic	8	circulatory system	13	13	13
455	Hemorrhoids	8	circulatory system	89	89	89
456	Chronic venous insufficiency [CVI]	8	circulatory system	24	24	24
457	Encounter for long-term (current) use of anticoagulants, antithrombotics, aspirin	8	circulatory system	66	66	66
457.2	Encounter for long-term (current) use of antiplatelets/antithrombotics	8	circulatory system	1	1	1
457.3	Encounter for long-term (current) use of aspirin	8	circulatory system	42	42	42
458	Hypotension	8	circulatory system	70	70	70
458.1	Orthostatic hypotension	8	circulatory system	0	0	0
458.2	Iatrogenic hypotension	8	circulatory system	2	2	2
458.9	Hypotension NOS	8	circulatory system	48	48	48
459	Other disorders of circulatory system	8	circulatory system	31	31	31
459.1	Hemorrhage NOS	8	circulatory system	0	0	0
459.7	Blood vessel replaced	8	circulatory system	1	1	1
459.9	Circulatory disease NEC	8	circulatory system	24	24	24
464	Acute sinusitis	9	respiratory	696	696	696
465	Acute upper respiratory infections of multiple or unspecified sites	9	respiratory	1296	1296	1296
465.2	Acute pharyngitis	9	respiratory	747	747	747
465.4	Acute laryngitis and tracheitis	9	respiratory	30	30	30
470	Septal Deviations/Turbinate Hypertrophy	9	respiratory	45	45	45
471	Nasal polyps	9	respiratory	10	10	10
472	Chronic pharyngitis and nasopharyngitis	9	respiratory	28	28	28
473	Diseases of the larynx and vocal cords	9	respiratory	43	43	43
473.1	Chronic laryngitis	9	respiratory	1	1	1
473.3	Paralysis/spasm of vocal cords or larynx	9	respiratory	4	4	4
473.4	Voice disturbance	9	respiratory	34	34	34
474	Acute and chronic tonsillitis	9	respiratory	104	104	104
474.1	Acute tonsillitis	9	respiratory	66	66	66
474.2	Chronic tonsillitis and adenoiditis	9	respiratory	36	36	36
475	Chronic sinusitis	9	respiratory	456	456	456
475.9	Postnasal drip	9	respiratory	41	41	41
476	Allergic rhinitis	9	respiratory	695	695	695
477	Epistaxis or throat hemorrhage	9	respiratory	39	39	39
478	Throat pain	9	respiratory	11	11	11
479	Other upper respiratory disease	9	respiratory	250	250	250
480	Pneumonia	9	respiratory	191	191	191
480.1	Bacterial pneumonia	9	respiratory	11	11	11
480.11	Pneumococcal pneumonia	9	respiratory	4	4	4
480.12	Pseudomonal pneumonia	9	respiratory	0	0	0
480.13	MRSA pneumonia	9	respiratory	0	0	0
480.2	Viral pneumonia	9	respiratory	1	1	1
480.3	Pneumonia due to fungus (mycoses)	9	respiratory	2	2	2
480.5	Bronchopneumonia and lung abscess	9	respiratory	1	1	1
481	Influenza	9	respiratory	47	47	47
483	Acute bronchitis and bronchiolitis	9	respiratory	194	194	194
495	Asthma	9	respiratory	606	606	606
495.1	Chronic obstructive asthma	9	respiratory	16	16	16
495.11	Chronic obstructive asthma with exacerbation	9	respiratory	11	11	11
495.2	Asthma with exacerbation	9	respiratory	136	136	136
496	Chronic airway obstruction	9	respiratory	141	141	141
496.1	Emphysema	9	respiratory	33	33	33
496.2	Chronic bronchitis	9	respiratory	54	54	54
496.21	Obstructive chronic bronchitis	9	respiratory	32	32	32
496.3	Bronchiectasis	9	respiratory	8	8	8
497	Bronchitis	9	respiratory	468	468	468

498	Acute bronchospasm	9	respiratory	83	83	83
499	Cystic fibrosis	9	respiratory	1	1	1
500	Lung disease due to external agents	9	respiratory	4	4	4
500.1	Extrinsic allergic alveolitis	9	respiratory	0	0	0
500.2	Pneumoconiosis	9	respiratory	0	0	0
501	Pneumonitis due to inhalation of food or vomitus	9	respiratory	0	0	0
502	Postinflammatory pulmonary fibrosis	9	respiratory	12	12	12
503	Pulmonary congestion and hypostasis	9	respiratory	4	4	4
504	Other alveolar and parietoalveolar pneumonopathy	9	respiratory	3	3	3
504.1	Idiopathic fibrosing alveolitis	9	respiratory	3	3	3
505	Other pulmonary inflamation or edema	9	respiratory	3	3	3
506	Empyema and pneumothorax	9	respiratory	10	10	10
507	Pleurisy; pleural effusion	9	respiratory	16	16	16
508	Pulmonary collapse; interstitial and compensatory emphysema	9	respiratory	2	2	2
509	Respiratory failure, insufficiency, arrest	9	respiratory	89	89	89
509.1	Respiratory failure	9	respiratory	63	63	63
509.2	Respiratory insufficiency	9	respiratory	0	0	0
509.3	Pulmonary insufficiency or respiratory failure following trauma and surgery	9	respiratory	5	5	5
509.5	Respiratory arrest	9	respiratory	0	0	0
509.8	Dependence on respirator [Ventilator] or supplemental oxygen	9	respiratory	33	33	33
510	Other diseases of lung	9	respiratory	36	36	36
510.2	Lung transplant	9	respiratory	0	0	0
512	Other symptoms of respiratory system	9	respiratory	1436	1436	1436
512.1	Wheezing	9	respiratory	95	95	95
512.2	Painful respiration	9	respiratory	94	94	94
512.3	Abnormal chest sounds	9	respiratory	10	10	10
512.7	Shortness of breath	9	respiratory	314	314	314
512.8	Cough	9	respiratory	785	785	785
512.9	Other dyspnea	9	respiratory	337	337	337
513	Respiratory abnormalities	9	respiratory	29	29	29
513.3	Hypoventilation	9	respiratory	14	14	14
513.31	Apnea	9	respiratory	5	5	5
513.32	Orthopnea	9	respiratory	2	2	2
513.4	Hyperventilation	9	respiratory	3	3	3
513.8	Disorders of diaphragm	9	respiratory	12	12	12
514	Abnormal findings examination of lungs	9	respiratory	71	71	71
514.1	Abnormal results of function study of pulmonary system	9	respiratory	0	0	0
514.2	Solitary pulmonary nodule	9	respiratory	85	85	85
516	Abnormal sputum	9	respiratory	12	12	12
516.1	Hemoptysis	9	respiratory	12	12	12
519	Other diseases of respiratory system, not elsewhere classified	9	respiratory	170	170	170
519.1	Tracheostomy complications	9	respiratory	1	1	1
519.2	Respiratory complications	9	respiratory	0	0	0
519.8	Other diseases of respiratory system, NEC	9	respiratory	147	147	147
519.9	Symptoms involving respiratory system and other chest symptoms	9	respiratory	18	18	18
520	Disorders of tooth development	10	digestive	0	0	0
520.1	Hereditary disturbances in tooth structure	10	digestive	0	0	0
520.2	Disturbances in tooth eruption	10	digestive	0	0	0
521	Diseases of hard tissues of teeth	10	digestive	12	12	12
521.1	Dental caries	10	digestive	5	5	5
521.2	Dental abrasion, erosion and attrition	10	digestive	1	1	1
521.4	Tooth complications likely association with other diseases	10	digestive	0	0	0
522	Diseases of pulp and periapical tissues	10	digestive	24	24	24
522.1	Pulpitis and necrosis of tooth pulp	10	digestive	0	0	0
522.5	Periapical abscess	10	digestive	24	24	24
523	Gingival and periodontal diseases	10	digestive	14	14	14
523.1	Gingivitis	10	digestive	3	3	3
523.3	Periodontitis (acute or chronic)	10	digestive	8	8	8
523.31	Acute periodontitis	10	digestive	8	8	8
523.32	Chronic periodontitis	10	digestive	0	0	0
524	Dentofacial anomalies, including malocclusion	10	digestive	2	2	2
524.3	Anomalies of tooth position/malocclusion	10	digestive	0	0	0
525	Other diseases of the teeth and supporting structures	10	digestive	37	37	37
525.1	Loss of teeth or edentulism	10	digestive	7	7	7
525.2	Atrophy of edentulous alveolar ridge	10	digestive	0	0	0
526	Diseases of the jaws	10	digestive	52	52	52
526.1	Cysts of the jaws	10	digestive	0	0	0

526.3	Anomalies of jaw size/symmetry	10	digestive	1	1	1
526.4	Temporomandibular joint disorders	10	digestive	37	37	37
526.41	Temporomandibular joint disorder, unspecified	10	digestive	1	1	1
526.42	Arthralgia/ankylosis of temporomandibular joint	10	digestive	10	10	10
526.5	Inflammatory conditions of jaw	10	digestive	1	1	1
526.8	Exostosis of jaw	10	digestive	0	0	0
526.9	Jaw disease NOS	10	digestive	0	0	0
527	Diseases of the salivary glands	10	digestive	26	26	26
527.1	Hypertrophy of salivary gland	10	digestive	1	1	1
527.2	Sialoadenitis	10	digestive	11	11	11
527.7	Disturbance of salivary secretion	10	digestive	12	12	12
527.8	Other specified diseases of the salivary glands	10	digestive	2	2	2
528	Diseases of the oral soft tissues, excluding lesions specific for gingiva and tongue	10	digestive	61	61	61
528.1	Stomatitis and mucositis	10	digestive	18	18	18
528.11	Stomatitis and mucositis (ulcerative)	10	digestive	0	0	0
528.12	Oral aphthae	10	digestive	14	14	14
528.3	Cellulitis and abscess of oral soft tissues	10	digestive	2	2	2
528.4	Cysts of oral soft tissues	10	digestive	0	0	0
528.41	Cyst of the salivary gland	10	digestive	0	0	0
528.5	Diseases of lips	10	digestive	9	9	9
528.6	Leukoplakia of oral mucosa	10	digestive	2	2	2
528.7	Sialolithiasis	10	digestive	1	1	1
529	Diseases and other conditions of the tongue	10	digestive	19	19	19
529.1	Glossitis	10	digestive	1	1	1
529.6	Glossodynia	10	digestive	7	7	7
530	Diseases of esophagus	10	digestive	777	777	777
530.1	Esophagitis, GERD and related diseases	10	digestive	753	753	753
530.11	GERD	10	digestive	676	676	676
530.12	Ulcer of esophagus	10	digestive	0	0	0
530.13	Barrett's esophagus	10	digestive	34	34	34
530.14	Reflux esophagitis	10	digestive	91	91	91
530.15	Eosinophilic esophagitis	10	digestive	10	10	10
530.2	Esophageal bleeding (varices/hemorrhage)	10	digestive	5	5	5
530.3	Stricture and stenosis of esophagus	10	digestive	11	11	11
530.5	Disorders of esophageal motility	10	digestive	8	8	8
530.6	Diverticulum of esophagus, acquired	10	digestive	0	0	0
530.7	Gastroesophageal laceration-hemorrhage syndrome	10	digestive	0	0	0
530.9	Heartburn	10	digestive	54	54	54
531	Peptic ulcer (excl. esophageal)	10	digestive	35	35	35
531.1	Hemorrhage from gastrointestinal ulcer	10	digestive	2	2	2
531.2	Gastric ulcer	10	digestive	6	6	6
531.3	Duodenal ulcer	10	digestive	0	0	0
531.4	Peptic ulcer, site unspecified	10	digestive	27	27	27
531.5	Gastrojejunal ulcer	10	digestive	0	0	0
532	Dysphagia	10	digestive	122	122	122
535	Gastritis and duodenitis	10	digestive	62	62	62
535.1	Acute gastritis	10	digestive	0	0	0
535.2	Atrophic gastritis	10	digestive	4	4	4
535.6	Duodenitis	10	digestive	3	3	3
535.8	Other specified gastritis	10	digestive	1	1	1
535.9	Gastritis and duodenitis, NOS	10	digestive	42	42	42
536	Disorders of function of stomach	10	digestive	104	104	104
536.3	Gastroparesis	10	digestive	13	13	13
536.7	Complications of gastrostomy, colostomy and enterostomy	10	digestive	1	1	1
536.8	Dyspepsia and other specified disorders of function of stomach	10	digestive	89	89	89
537	Other disorders of stomach and duodenum	10	digestive	5	5	5
537.1	Lesions of stomach and duodenum	10	digestive	1	1	1
539	Bariatric surgery	10	digestive	83	83	83
540	Appendiceal conditions	10	digestive	36	36	36
540.1	Appendicitis	10	digestive	35	35	35
540.11	Acute appendicitis	10	digestive	22	22	22
550	Abdominal hernia	10	digestive	256	256	256
550.1	Inguinal hernia	10	digestive	60	60	60
550.2	Diaphragmatic hernia	10	digestive	103	103	103
550.3	Femoral hernia	10	digestive	0	0	0
550.4	Umbilical hernia	10	digestive	39	39	39
550.5	Ventral hernia	10	digestive	25	25	25

550.6	Incisional hernia	10	digestive	18	18	18
555	Inflammatory bowel disease and other gastroenteritis and colitis	10	digestive	57	57	57
555.1	Regional enteritis	10	digestive	29	29	29
555.2	Ulcerative colitis	10	digestive	31	31	31
555.21	Ulcerative colitis (chronic)	10	digestive	9	9	9
556	Ulceration of the lower GI tract	10	digestive	4	4	4
556.1	Ulceration of intestine	10	digestive	4	4	4
556.11	Angiodysplasia of intestine (without mention of hemorrhage)	10	digestive	4	4	4
557	Intestinal malabsorption (non-celiac)	10	digestive	14	14	14
557.1	Celiac disease	10	digestive	0	0	0
558	Noninfectious gastroenteritis	10	digestive	158	158	158
559	Ileostomy status	10	digestive	7	7	7
560	Intestinal obstruction without mention of hernia	10	digestive	47	47	47
560.1	Paralytic ileus	10	digestive	13	13	13
560.2	Impaction of intestine	10	digestive	2	2	2
560.3	Peritoneal or intestinal adhesions	10	digestive	2	2	2
560.4	Other intestinal obstruction	10	digestive	30	30	30
561	Symptoms involving digestive system	10	digestive	43	43	43
562	Diverticulosis and diverticulitis	10	digestive	158	158	158
562.1	Diverticulosis	10	digestive	20	20	20
562.2	Diverticulitis	10	digestive	80	80	80
564	Functional digestive disorders	10	digestive	209	209	209
564.1	Irritable Bowel Syndrome	10	digestive	93	93	93
564.9	Personal history of diseases of digestive system	10	digestive	1	1	1
565	Anal and rectal conditions	10	digestive	34	34	34
565.1	Anal and rectal polyp	10	digestive	0	0	0
567	Peritonitis and retroperitoneal infections	10	digestive	8	8	8
568	Other disorders of peritoneum	10	digestive	7	7	7
568.1	Peritoneal adhesions (postoperative) (postinfection)	10	digestive	0	0	0
569	Other disorders of intestine	10	digestive	29	29	29
569.1	Toxic gastroenteritis and colitis	10	digestive	0	0	0
569.2	Gastrointestinal complications	10	digestive	5	5	5
571	Chronic liver disease and cirrhosis	10	digestive	98	98	98
571.5	Other chronic nonalcoholic liver disease	10	digestive	91	91	91
571.51	Cirrhosis of liver without mention of alcohol	10	digestive	9	9	9
571.6	Primary biliary cirrhosis	10	digestive	5	5	5
571.8	Liver abscess and sequelae of chronic liver disease	10	digestive	8	8	8
571.81	Portal hypertension	10	digestive	4	4	4
572	Ascites (non malignant)	10	digestive	6	6	6
573	Other disorders of liver	10	digestive	40	40	40
573.1	Chronic passive congestion of liver	10	digestive	0	0	0
573.2	Liver replaced by transplant	10	digestive	2	2	2
573.3	Hepatomegaly	10	digestive	11	11	11
573.4	Acute and subacute necrosis of liver	10	digestive	3	3	3
573.5	Jaundice (not of newborn)	10	digestive	9	9	9
573.6	Nonspecific elevation of levels of transaminase or lactic acid dehydrogenase [LDH]	10	digestive	71	71	71
573.7	Abnormal results of function study of liver	10	digestive	19	19	19
573.9	Abnormal serum enzyme levels	10	digestive	110	110	110
574	Cholelithiasis and cholecystitis	10	digestive	122	122	122
574.1	Cholelithiasis	10	digestive	88	88	88
574.11	Cholelithiasis with acute cholecystitis	10	digestive	1	1	1
574.12	Cholelithiasis with other cholecystitis	10	digestive	6	6	6
574.2	Calculus of bile duct	10	digestive	16	16	16
574.3	Cholecystitis without cholelithiasis	10	digestive	9	9	9
575	Other biliary tract disease	10	digestive	61	61	61
575.1	Cholangitis	10	digestive	1	1	1
575.2	Obstruction of bile duct	10	digestive	1	1	1
575.6	Cholesterolosis of gallbladder	10	digestive	7	7	7
575.7	Other disorders of gallbladder	10	digestive	26	26	26
575.8	Other disorders of biliary tract	10	digestive	6	6	6
575.9	Nonspecific abnormal findings on radiological and other examination of biliary tract	10	digestive	5	5	5
577	Diseases of pancreas	10	digestive	57	57	57
577.1	Acute pancreatitis	10	digestive	11	11	11
577.2	Chronic pancreatitis	10	digestive	3	3	3
577.3	Cyst and pseudocyst of pancreas	10	digestive	11	11	11
578	Gastrointestinal hemorrhage	10	digestive	128	128	128
578.1	Hematemesis	10	digestive	0	0	0

578.2	Blood in stool	10	digestive	45	45	45
578.8	Hemorrhage of rectum and anus	10	digestive	48	48	48
578.9	Hemorrhage of gastrointestinal tract	10	digestive	33	33	33
579	Other symptoms involving abdomen and pelvis	10	digestive	327	327	327
579.2	Splenomegaly	10	digestive	15	15	15
579.8	Nonspecific abnormal findings in stool contents	10	digestive	23	23	23
580	Nephritis; nephrosis; renal sclerosis	11	genitourinary	17	17	17
580.1	Glomerulonephritis	11	genitourinary	1	1	1
580.11	Proliferative glomerulonephritis	11	genitourinary	0	0	0
580.12	Non-proliferative glomerulonephritis	11	genitourinary	1	1	1
580.13	Acute glomerulonephritis, NOS	11	genitourinary	0	0	0
580.14	Chronic glomerulonephritis, NOS	11	genitourinary	0	0	0
580.2	Nephrotic syndrome without mention of glomerulonephritis	11	genitourinary	0	0	0
580.3	Nephritis and nephropathy without mention of glomerulonephritis	11	genitourinary	14	14	14
580.31	Nephritis and nephropathy in diseases classified elsewhere	11	genitourinary	11	11	11
580.32	Nephritis and nephropathy with pathological lesion	11	genitourinary	2	2	2
580.4	Renal sclerosis, NOS	11	genitourinary	2	2	2
585	Renal failure	11	genitourinary	223	223	223
585.1	Acute renal failure	11	genitourinary	78	78	78
585.2	Renal failure NOS	11	genitourinary	6	6	6
585.3	Chronic renal failure [CKD]	11	genitourinary	141	141	141
585.31	Renal dialysis	11	genitourinary	1	1	1
585.32	End stage renal disease	11	genitourinary	2	2	2
585.33	Chronic Kidney Disease, Stage III	11	genitourinary	120	120	120
585.34	Chronic Kidney Disease, Stage IV	11	genitourinary	10	10	10
585.4	Chronic kidney disease, Stage I or II	11	genitourinary	42	42	42
586	Other disorders of the kidney and ureters	11	genitourinary	177	177	177
586.1	Anatomical abnormatilies of kidney and ureters	11	genitourinary	0	0	0
586.11	Small kidney	11	genitourinary	0	0	0
586.12	Vesicoureteral reflux	11	genitourinary	0	0	0
586.2	Cyst of kidney, acquired	11	genitourinary	27	27	27
586.3	Vascular disorders of kidney/hypertrophy	11	genitourinary	2	2	2
586.4	Stricture/obstruction of ureter	11	genitourinary	5	5	5
587	Kidney replaced by transpant	11	genitourinary	0	0	0
588	Disorders resulting from impaired renal function	11	genitourinary	4	4	4
588.1	Renal osteodystrophy	11	genitourinary	0	0	0
588.2	Secondary hyperparathyroidism (of renal origin)	11	genitourinary	4	4	4
589	Abnormal results of function study of kidney	11	genitourinary	25	25	25
590	Pyelonephritis	11	genitourinary	52	52	52
591	Urinary tract infection	11	genitourinary	87	87	87
592	Cystitis and urethritis	11	genitourinary	214	214	214
592.1	Cystitis	11	genitourinary	72	72	72
592.11	Acute cystitis	11	genitourinary	16	16	16
592.12	Chronic cystitis	11	genitourinary	1	1	1
592.13	Chronic interstitial cystitis	11	genitourinary	13	13	13
592.2	Urethritis and urethral syndrome	11	genitourinary	12	12	12
592.21	Urethral syndrome	11	genitourinary	7	7	7
592.3	Urethral stricture due to infecton	11	genitourinary	0	0	0
593	Hematuria	11	genitourinary	174	174	174
593.1	Gross hematuria	11	genitourinary	21	21	21
593.2	Microscopic hematuria	11	genitourinary	40	40	40
594	Urinary calculus	11	genitourinary	217	217	217
594.1	Calculus of kidney	11	genitourinary	14	14	14
594.2	Calculus of lower urinary tract	11	genitourinary	2	2	2
594.3	Calculus of ureter	11	genitourinary	51	51	51
594.8	Renal colic	11	genitourinary	0	0	0
595	Hydronephrosis	11	genitourinary	28	28	28
596	Other disorders of bladder	11	genitourinary	53	53	53
596.1	Bladder neck obstruction	11	genitourinary	0	0	0
596.5	Functional disorders of bladder	11	genitourinary	31	31	31
597	Other disorders of urethra and urinary tract	11	genitourinary	503	503	503
597.1	Urethral stricture (not specified as infectious)	11	genitourinary	1	1	1
597.2	Urinary complications NEC	11	genitourinary	1	1	1
597.8	Urethral hypermobility/ISD	11	genitourinary	0	0	0
598	Abnormal findings on examination of urine	11	genitourinary	26	26	26
598.4	Other cells and casts in urine	11	genitourinary	8	8	8
599	Other symptoms/disorders or the urinary system	11	genitourinary	657	657	657

599.1	Urinary obstruction	11	genitourinary	12	12	12
599.2	Retention of urine	11	genitourinary	48	48	48
599.3	Dysuria	11	genitourinary	254	254	254
599.4	Urinary incontinence	11	genitourinary	97	97	97
599.5	Frequency of urination and polyuria	11	genitourinary	142	142	142
599.6	Oliguria and anuria	11	genitourinary	0	0	0
599.7	Urethral discharge	11	genitourinary	4	4	4
599.8	Other symptoms involving urinary system	11	genitourinary	52	52	52
599.9	Other abnormality of urination	11	genitourinary	55	55	55
600	Hyperplasia of prostate	11	genitourinary	170	170	170
601	Inflammatory diseases of prostate	11	genitourinary	23	23	23
601.1	Prostatitis	11	genitourinary	4	4	4
601.11	Acute prostatitis	11	genitourinary	0	0	0
601.12	Chronic prostatitis	11	genitourinary	1	1	1
601.3	Orchitis and epididymitis	11	genitourinary	9	9	9
601.4	Balanoposthitis	11	genitourinary	4	4	4
601.8	Other inflammatory disorders of male genital organs	11	genitourinary	1	1	1
602	Other disorders of prostate	11	genitourinary	4	4	4
602.3	Dysplasia of prostate	11	genitourinary	0	0	0
603	Other disorders of testis	11	genitourinary	12	12	12
603.1	Hydrocele	11	genitourinary	8	8	8
603.2	Spermatocele	11	genitourinary	2	2	2
604	Disorders of penis	11	genitourinary	9	9	9
604.1	Redundant prepuce and phimosis/BXO	11	genitourinary	0	0	0
604.2	Vascular disorders of penis	11	genitourinary	0	0	0
604.3	Peyronie's disease	11	genitourinary	2	2	2
605	Erectile dysfunction [ED]	11	genitourinary	113	113	113
608	Other disorders of male genital organs	11	genitourinary	30	30	30
609	Male infertility and abnormal spermatozoa	11	genitourinary	3	3	3
609.1	Infertility, male	11	genitourinary	1	1	1
609.11	Azoospermia and oligospermia	11	genitourinary	0	0	0
609.2	Abnormal spermatozoa	11	genitourinary	2	2	2
610	Benign mammary dysplasias	11	genitourinary	78	78	78
610.1	Cystic mastopathy	11	genitourinary	35	35	35
610.2	Fibroadenosis of breast	11	genitourinary	1	1	1
610.3	Fibrosclerosis of breast	11	genitourinary	1	1	1
610.4	Benign neoplasm of breast	11	genitourinary	9	9	9
610.8	Other specified benign mammary dysplasias	11	genitourinary	6	6	6
611	Abnormal findings on mammogram or breast exam	11	genitourinary	574	574	574
611.1	Abnormal mammogram	11	genitourinary	249	249	249
611.11	Mammographic microcalcification	11	genitourinary	22	22	22
611.3	Lump or mass in breast	11	genitourinary	204	204	204
612	Breast conditions, congenital or relating to hormones	11	genitourinary	31	31	31
612.1	Galactorrhea	11	genitourinary	4	4	4
612.2	Hypertrophy of breast (Gynecomastia)	11	genitourinary	23	23	23
612.3	Congenital anomalies of breast	11	genitourinary	0	0	0
613	Other nonmalignant breast conditions	11	genitourinary	180	180	180
613.1	Inflammatory disease of breast	11	genitourinary	1	1	1
613.5	Mastodynia	11	genitourinary	97	97	97
613.7	Other signs and symptoms in breast	11	genitourinary	28	28	28
613.8	Other specified disorders of breast	11	genitourinary	11	11	11
613.9	Breast disorder NOS	11	genitourinary	10	10	10
614	Inflammatory diseases of female pelvic organs	11	genitourinary	178	178	178
614.1	Pelvic peritoneal adhesions, female (postoperative) (postinfection)	11	genitourinary	0	0	0
614.3	Pelvic inflammatory disease (PID)	11	genitourinary	5	5	5
614.31	Acute inflammatory pelvic disease	11	genitourinary	3	3	3
614.32	Chronic inflammatory pelvic disease	11	genitourinary	0	0	0
614.33	Pelvic inflammatory disease, NOS	11	genitourinary	1	1	1
614.4	Inflammatory diseases of uterus, except cervix	11	genitourinary	3	3	3
614.5	Inflammatory disease of cervix, vagina, and vulva	11	genitourinary	167	167	167
614.51	Cervicitis and endocervicitis	11	genitourinary	0	0	0
614.52	Vaginitis and vulvovaginitis	11	genitourinary	146	146	146
614.53	Cyst or abscess of Bartholin's gland	11	genitourinary	4	4	4
614.54	Abscess or ulceration of vulva	11	genitourinary	8	8	8
615	Endometriosis	11	genitourinary	41	41	41
618	Genital prolapse	11	genitourinary	49	49	49
618.1	Prolapse of vaginal walls	11	genitourinary	30	30	30

618.2	Uterine/Uterovaginal prolapse	11	genitourinary	4	4	4
618.5	Prolapse of vaginal vault after hysterectomy	11	genitourinary	0	0	0
618.6	Vaginal enterocele, congenital or acquired	11	genitourinary	0	0	0
619	Noninflammatory female genital disorders	11	genitourinary	133	133	133
619.1	Noninflammatory disorders of ovary, fallopian tube, and broad ligament	11	genitourinary	5	5	5
619.2	Disorders of uterus, NEC	11	genitourinary	9	9	9
619.3	Noninflammatory disorders of cervix	11	genitourinary	2	2	2
619.4	Noninflammatory disorders of vagina	11	genitourinary	31	31	31
619.5	Noninflammatory disorders of vulva and perineum	11	genitourinary	7	7	7
620	Dysplasia of female genital organs	11	genitourinary	6	6	6
620.1	Dysplasia of cervix	11	genitourinary	3	3	3
621	Endometrial hyperplasia	11	genitourinary	6	6	6
622	Polyp of female genital organs	11	genitourinary	6	6	6
622.1	Polyp of corpus uteri	11	genitourinary	0	0	0
622.2	Mucous polyp of cervix	11	genitourinary	5	5	5
623	Hypertrophy of female genital organs	11	genitourinary	14	14	14
624	Symptoms involving female genital tract	11	genitourinary	36	36	36
624.1	Dystrophy of female genital tract	11	genitourinary	1	1	1
624.2	Atrophy of female genital tract	11	genitourinary	1	1	1
624.9	stress incontinence, female	11	genitourinary	5	5	5
625	Pain and other symptoms associated with female genital organs	11	genitourinary	208	208	208
625.1	Dyspareunia	11	genitourinary	2	2	2
626	Disorders of menstruation and other abnormal bleeding from female genital tract	11	genitourinary	435	435	435
626.1	Irregular menstrual cycle/bleeding	11	genitourinary	315	315	315
626.11	Absent or infrequent menstruation	11	genitourinary	16	16	16
626.12	Excessive or frequent menstruation	11	genitourinary	111	111	111
626.13	Irregular menstrual cycle	11	genitourinary	48	48	48
626.14	Irregular menstrual bleeding	11	genitourinary	17	17	17
626.15	Infertility, female, associated with anovulation	11	genitourinary	0	0	0
626.2	Dysmenorrhea	11	genitourinary	47	47	47
626.21	Mittelschmerz	11	genitourinary	3	3	3
626.4	Premenstrual tension syndromes	11	genitourinary	15	15	15
626.8	Infertility, female	11	genitourinary	23	23	23
627	Menopausal and postmenopausal disorders	11	genitourinary	419	419	419
627.1	Postmenopausal bleeding	11	genitourinary	35	35	35
627.2	Symptomatic menopause	11	genitourinary	269	269	269
627.21	Symptomatic artificial menopause	11	genitourinary	3	3	3
627.22	Need for Hormone replacement therapy (postmenopausal)	11	genitourinary	92	92	92
627.3	Postmenopausal atrophic vaginitis	11	genitourinary	56	56	56
627.4	Premenopausal menorrhagia	11	genitourinary	0	0	0
627.5	Premature menopause and other ovarian failure	11	genitourinary	10	10	10
628	Ovarian cyst	11	genitourinary	122	122	122
634	Miscarriage; stillbirth	12	pregnancy complications	34	34	34
634.1	Missed abortion/Hydatidiform mole	12	pregnancy complications	4	4	4
634.3	Ectopic pregnancy	12	pregnancy complications	7	7	7
635	Hemorrhage during pregnancy; childbirth and postpartum	12	pregnancy complications	11	11	11
635.2	Antepartum hemorrhage, abruptio placentae, and placenta previa	12	pregnancy complications	11	11	11
635.3	Placenta previa and abruptio placenta	12	pregnancy complications	0	0	0
636	Early or threatened labor; hemorrhage in early pregnancy	12	pregnancy complications	82	82	82
636.1	Threatened premature labor	12	pregnancy complications	2	2	2
636.2	Early onset of delivery	12	pregnancy complications	4	4	4
636.3	Hemorrhage in early pregnancy	12	pregnancy complications	44	44	44
636.8	Cervical incompetence	12	pregnancy complications	3	3	3
637	Short gestation; low birth weight; and fetal growth retardation	12	pregnancy complications	0	0	0
642	Hypertension complicating pregnancy, childbirth, and the puerperium	12	pregnancy complications	32	32	32
642.1	Preeclampsia and eclampsia	12	pregnancy complications	9	9	9
643	Excessive vomiting in pregnancy	12	pregnancy complications	12	12	12
643.1	Hyperemesis gravidarum	12	pregnancy complications	1	1	1
644	Anemia during pregnancy	12	pregnancy complications	3	3	3
646	Other complications of pregnancy NEC	12	pregnancy complications	29	29	29
647	Infectious and parasitic complications affecting pregnancy	12	pregnancy complications	6	6	6
647.1	Infections of genitourinary tract during pregnancy	12	pregnancy complications	3	3	3
647.3	Major puerperal infection	12	pregnancy complications	0	0	0
649	Other conditions or status of the mother complicating pregnancy, childbirth, or the puerperium	12	pregnancy complications	46	46	46
649.1	Diabetes or abnormal glucose tolerance complicating pregnancy	12	pregnancy complications	27	27	27
653	Problems associated with amniotic cavity and membranes	12	pregnancy complications	18	18	18
654	Other and unspecified complications of birth; puerperium affecting management of mother	12	pregnancy complications	0	0	0

654.1	Abnormality of organs and soft tissues of pelvis complicating pregnancy, childbirth, or the puerperium	12	pregnancy complications	4	4	4
654.2	Rhesus isoimmunization in pregnancy	12	pregnancy complications	4	4	4
655	Known or suspected fetal abnormality affecting management of mother	12	pregnancy complications	75	75	75
655.1	Abnormality in fetal heart rate or rhythm	12	pregnancy complications	1	1	1
656	Other perinatal conditions of fetus or newborn	12	pregnancy complications	0	0	0
656.1	Isoimmunization of fetus or newborn	12	pregnancy complications	0	0	0
656.2	Respiratory conditions of fetus and newborn	12	pregnancy complications	0	0	0
656.22	Interstitial emphysema and related conditions of newborn	12	pregnancy complications	0	0	0
656.26	Transitory tachypnea or apnea of newborn	12	pregnancy complications	0	0	0
656.3	Endocrine and metabolic disturbances of fetus and newborn	12	pregnancy complications	0	0	0
656.4	Hemorrhage of fetus or newborn	12	pregnancy complications	0	0	0
656.5	Hematological disorders of newborn	12	pregnancy complications	0	0	0
656.6	Perinatal disorders of digestive system	12	pregnancy complications	0	0	0
656.7	Conditions involving the integument and temperature regulation of fetus and newborn	12	pregnancy complications	0	0	0
656.8	Perinatal jaundice	12	pregnancy complications	0	0	0
656.9	Neonatal bradycardia or tachycardia	12	pregnancy complications	0	0	0
657	Infections specific to the perinatal period	12	pregnancy complications	0	0	0
669	Complications of labor and delivery NEC	12	pregnancy complications	2	2	2
671	Venous/cerebrovascular complications embolism in pregnancy and the puerperium	12	pregnancy complications	11	11	11
674	Other complications of the puerperium NEC	12	pregnancy complications	0	0	0
676	Other disorders of the breast associated with childbirth and disorders of lactation	12	pregnancy complications	2	2	2
681	Superficial cellulitis and abscess	13	dermatologic	282	282	282
681.1	Cellulitis and abscess of fingers/toes	13	dermatologic	35	35	35
681.2	Cellulitis and abscess of face/neck	13	dermatologic	8	8	8
681.3	Cellulitis and abscess of arm/hand	13	dermatologic	22	22	22
681.5	Cellulitis and abscess of leg, except foot	13	dermatologic	44	44	44
681.6	Cellulitis and abscess of foot, toe	13	dermatologic	12	12	12
681.7	Cellulitis and abscess of trunk	13	dermatologic	55	55	55
686	Other local infections of skin and subcutaneous tissue	13	dermatologic	67	67	67
686.1	Carbuncle and furuncle	13	dermatologic	11	11	11
686.2	Impetigo	13	dermatologic	6	6	6
686.3	Pilonidal cyst	13	dermatologic	4	4	4
686.4	Pyogenic granuloma	13	dermatologic	0	0	0
686.5	Pyoderma	13	dermatologic	0	0	0
687	Symptoms affecting skin	13	dermatologic	51	51	51
687.1	Rash and other nonspecific skin eruption	13	dermatologic	224	224	224
687.3	Changes in skin texture	13	dermatologic	16	16	16
687.4	Disturbance of skin sensation	13	dermatologic	59	59	59
689	Disorder of skin and subcutaneous tissue NOS	13	dermatologic	243	243	243
690	Erythematosquamous dermatosis	13	dermatologic	11	11	11
690.1	Seborrheic dermatitis	13	dermatologic	11	11	11
691	Congenital anomalies of skin	13	dermatologic	4	4	4
691.1	Ichthyosis congenita	13	dermatologic	0	0	0
691.3	Congenital pigmentary anomalies of skin	13	dermatologic	1	1	1
694	Dyschromia and Vitiligo	13	dermatologic	29	29	29
694.1	Vitiligo	13	dermatologic	4	4	4
694.2	Other dyschromia	13	dermatologic	23	23	23
694.3	Vascular disorders of skin	13	dermatologic	2	2	2
695	Erythematous conditions	13	dermatologic	94	94	94
695.1	Toxic erythema	13	dermatologic	3	3	3
695.2	Bullous dermatoses	13	dermatologic	2	2	2
695.21	Dermatitis herpetiformis	13	dermatologic	0	0	0
695.22	Pemphigus and pemphigoid	13	dermatologic	2	2	2
695.3	Rosacea	13	dermatologic	43	43	43
695.4	Lupus (localized and systemic)	13	dermatologic	21	21	21
695.41	Cutaneous lupus erythematosus	13	dermatologic	6	6	6
695.42	Systemic lupus erythematosus	13	dermatologic	10	10	10
695.7	Prurigo and Lichen	13	dermatologic	10	10	10
695.8	Other specified erythematous conditions	13	dermatologic	10	10	10
695.81	Erythema nodosum	13	dermatologic	3	3	3
695.9	Unspecified erythematous condition	13	dermatologic	3	3	3
696	Psoriasis and related disorders	13	dermatologic	46	46	46
696.2	Parapsoriasis	13	dermatologic	0	0	0
696.3	Pityriasis	13	dermatologic	0	0	0
696.4	Psoriasis	13	dermatologic	42	42	42
696.41	Psoriasis vulgaris	13	dermatologic	40	40	40
696.42	Psoriatic arthropathy	13	dermatologic	1	1	1

697	Sarcoidosis	13	dermatologic	13	13	13
698	Pruritus and related conditions	13	dermatologic	80	80	80
700	Corns and callosities	13	dermatologic	14	14	14
701	Other hypertrophic and atrophic conditions of skin	13	dermatologic	96	96	96
701.1	Keratoderma, acquired	13	dermatologic	1	1	1
701.2	Scar conditions and fibrosis of skin	13	dermatologic	5	5	5
701.3	Circumscribed scleroderma	13	dermatologic	0	0	0
701.4	Keloid scar	13	dermatologic	3	3	3
701.5	Abnormal granulation tissue	13	dermatologic	1	1	1
701.6	Acquired acanthosis nigricans	13	dermatologic	2	2	2
702	Degenerative skin conditions and other dermatoses	13	dermatologic	141	141	141
702.1	Actinic keratosis	13	dermatologic	0	0	0
702.2	Seborrheic keratosis	13	dermatologic	62	62	62
702.4	Degenerative skin disorders	13	dermatologic	0	0	0
703	Diseases of nail, NOS	13	dermatologic	46	46	46
703.1	Ingrowing nail	13	dermatologic	1	1	1
704	Diseases of hair and hair follicles	13	dermatologic	105	105	105
704.1	Alopecia	13	dermatologic	13	13	13
704.11	Alopecia Areata	13	dermatologic	1	1	1
704.12	Telogen effluvium	13	dermatologic	0	0	0
704.2	Hirsutism	13	dermatologic	13	13	13
704.8	Other specified diseases of hair and hair follicles	13	dermatologic	9	9	9
705	Disorders of sweat glands	13	dermatologic	16	16	16
705.1	Dyshidrosis	13	dermatologic	8	8	8
705.3	Hidradenitis	13	dermatologic	5	5	5
705.8	Hyperhidrosis	13	dermatologic	34	34	34
706	Diseases of sebaceous glands	13	dermatologic	153	153	153
706.1	Acne	13	dermatologic	96	96	96
706.2	Sebaceous cyst	13	dermatologic	53	53	53
706.3	Seborrhea	13	dermatologic	0	0	0
706.8	Other specified diseases of sebaceous glands	13	dermatologic	1	1	1
707	Chronic ulcer of skin	13	dermatologic	27	27	27
707.1	Decubitus ulcer	13	dermatologic	8	8	8
707.2	Chronic ulcer of leg or foot	13	dermatologic	15	15	15
707.3	Chronic ulcer of unspecified site	13	dermatologic	8	8	8
709	Diffuse diseases of connective tissue	13	dermatologic	40	40	40
709.2	Sicca syndrome	13	dermatologic	20	20	20
709.3	Systemic sclerosis	13	dermatologic	3	3	3
709.4	Polymyositis	13	dermatologic	1	1	1
709.5	Dermatomyositis	13	dermatologic	0	0	0
709.6	Other specified diffuse diseases of connective tissue	13	dermatologic	1	1	1
709.7	Unspecified diffuse connective tissue disease	13	dermatologic	6	6	6
710	Osteomyelitis, periostitis, and other infections involving bone	14	musculoskeletal	19	19	19
710.1	Osteomyelitis	14	musculoskeletal	19	19	19
710.11	Acute osteomyelitis	14	musculoskeletal	3	3	3
710.12	Chronic osteomyelitis	14	musculoskeletal	2	2	2
710.19	Unspecified osteomyelitis	14	musculoskeletal	14	14	14
710.2	Periostitis	14	musculoskeletal	0	0	0
710.3	Osteopathy resulting from poliomyelitis	14	musculoskeletal	0	0	0
711	Arthropathy associated with infections	14	musculoskeletal	8	8	8
711.1	Pyogenic arthritis	14	musculoskeletal	2	2	2
711.2	Reiter's disease	14	musculoskeletal	2	2	2
711.3	Behcet's syndrome	14	musculoskeletal	0	0	0
712	Infective connective tissue disorders	14	musculoskeletal	1	1	1
713	Arthropathy associated with other disorders classified elsewhere	14	musculoskeletal	3	3	3
713.5	Arthropathy associated with neurological disorders	14	musculoskeletal	0	0	0
714	Rheumatoid arthritis and other inflammatory polyarthropathies	14	musculoskeletal	73	73	73
714.1	Rheumatoid arthritis	14	musculoskeletal	37	37	37
714.2	Juvenile rheumatoid arthritis	14	musculoskeletal	1	1	1
715	Other inflammatory spondylopathies	14	musculoskeletal	34	34	34
715.1	Sacroiliitis NEC	14	musculoskeletal	12	12	12
715.2	Ankylosing spondylitis	14	musculoskeletal	2	2	2
715.3	Spinal enthesopathy	14	musculoskeletal	3	3	3
716	Other arthropathies	14	musculoskeletal	268	268	268
716.1	Unspecified polyarthropathy or polyarthritis	14	musculoskeletal	10	10	10
716.2	Unspecified monoarthritis	14	musculoskeletal	0	0	0
716.3	Kaschin-Beck disease	14	musculoskeletal	0	0	0

716.8	Palindromic rheumatism	14	musculoskeletal	0	0	0
716.9	Arthropathy NOS	14	musculoskeletal	245	245	245
717	Polymyalgia Rheumatica	14	musculoskeletal	5	5	5
720	Spinal stenosis	14	musculoskeletal	145	145	145
720.1	Spinal stenosis of lumbar region	14	musculoskeletal	126	126	126
721	Spondylosis and allied disorders	14	musculoskeletal	291	291	291
721.1	Spondylosis without myelopathy	14	musculoskeletal	217	217	217
721.2	Spondylosis with myelopathy	14	musculoskeletal	9	9	9
721.8	Other allied disorders of spine	14	musculoskeletal	5	5	5
722	Intervertebral disc disorders	14	musculoskeletal	519	519	519
722.1	Displacement of intervertebral disc	14	musculoskeletal	162	162	162
722.3	Schmorl's nodes	14	musculoskeletal	4	4	4
722.6	Degeneration of intervertebral disc	14	musculoskeletal	328	328	328
722.7	Intervertebral disc disorder with myelopathy	14	musculoskeletal	9	9	9
722.8	Postlaminectomy syndrome	14	musculoskeletal	15	15	15
722.9	Other and unspecified disc disorder	14	musculoskeletal	32	32	32
723	Other disorders of cervical region	14	musculoskeletal	48	48	48
723.1	Torticollis	14	musculoskeletal	14	14	14
724	Other and unspecified disorders of back	14	musculoskeletal	133	133	133
724.1	Disorders of sacrum	14	musculoskeletal	25	25	25
724.2	Disorders of coccyx	14	musculoskeletal	8	8	8
724.8	Other symptoms referable to back	14	musculoskeletal	82	82	82
724.9	Other unspecified back disorders	14	musculoskeletal	4	4	4
726	Peripheral enthesopathies and allied syndromes	14	musculoskeletal	371	371	371
726.1	Enthesopathy	14	musculoskeletal	172	172	172
726.2	Synoviopathy	14	musculoskeletal	22	22	22
726.3	Bursitis	14	musculoskeletal	39	39	39
726.4	Calcaneal spur; Exostosis NOS	14	musculoskeletal	7	7	7
727	Other disorders of synovium, tendon, and bursa	14	musculoskeletal	218	218	218
727.1	Synovitis and tenosynovitis	14	musculoskeletal	95	95	95
727.2	Bursitis disorders	14	musculoskeletal	4	4	4
727.4	Ganglion and cyst of synovium, tendon, and bursa	14	musculoskeletal	23	23	23
727.5	Rupture of synovium	14	musculoskeletal	17	17	17
727.6	Rupture of tendon, nontraumatic	14	musculoskeletal	26	26	26
727.7	Contracture of tendon (sheath)	14	musculoskeletal	1	1	1
727.8	Plica syndrome	14	musculoskeletal	1	1	1
728	Disorders of muscle, ligament, and fascia	14	musculoskeletal	3	3	3
728.1	Muscular calcification and ossification	14	musculoskeletal	0	0	0
728.2	Laxity of ligament or hypermobility syndrome	14	musculoskeletal	2	2	2
728.7	Fasciitis	14	musculoskeletal	79	79	79
728.71	Contracture of palmar fascia [Dupuytren's disease]	14	musculoskeletal	7	7	7
729	Other disorders of soft tissues	14	musculoskeletal	13	13	13
729.1	Rheumatism, unspecified and fibrositis	14	musculoskeletal	0	0	0
729.3	Panniculitis	14	musculoskeletal	2	2	2
729.7	Nontraumatic compartment syndrome	14	musculoskeletal	0	0	0
731	Osteitis deformans and osteopathies associated with other disorders classified elsewhere	14	musculoskeletal	0	0	0
731.1	Osteitis deformans [Paget's disease of bone]	14	musculoskeletal	0	0	0
732	Osteochondropathies	14	musculoskeletal	5	5	5
732.1	Juvenile osteochondrosis	14	musculoskeletal	1	1	1
732.7	Osteochondritis dissecans	14	musculoskeletal	1	1	1
733	Other disorders of bone and cartilage	14	musculoskeletal	383	383	383
733.2	Cyst of bone	14	musculoskeletal	6	6	6
733.4	Aseptic necrosis of bone	14	musculoskeletal	4	4	4
733.6	Costochondritis	14	musculoskeletal	27	27	27
733.8	Malunion and nonunion of fracture	14	musculoskeletal	6	6	6
733.9	Chondromalacia	14	musculoskeletal	4	4	4
735	Acquired foot deformities	14	musculoskeletal	95	95	95
735.1	Flat foot	14	musculoskeletal	8	8	8
735.2	Acquired toe deformities	14	musculoskeletal	26	26	26
735.21	Hammer toe (acquired)	14	musculoskeletal	15	15	15
735.22	Claw toe (acquired)	14	musculoskeletal	0	0	0
735.23	Hallux rigidus	14	musculoskeletal	5	5	5
735.3	Hallux valgus (Bunion)	14	musculoskeletal	32	32	32
736	Other acquired deformities of limbs	14	musculoskeletal	11	11	11
736.1	Acquired deformities of forearm	14	musculoskeletal	0	0	0
736.2	Acquired deformities of finger	14	musculoskeletal	3	3	3
736.3	Acquired deformities of hip	14	musculoskeletal	0	0	0

736.4	Genu valgum or varum (acquired)	14	musculoskeletal	3	3	3
736.5	Acquired deformities of knee	14	musculoskeletal	0	0	0
736.6	Unequal leg length (acquired)	14	musculoskeletal	3	3	3
737	Curvature of spine	14	musculoskeletal	77	77	77
737.1	Kyphosis (acquired)	14	musculoskeletal	9	9	9
737.2	Lordosis (acquired)	14	musculoskeletal	0	0	0
737.3	Kyphoscoliosis and scoliosis	14	musculoskeletal	60	60	60
738	Other acquired musculoskeletal deformity	14	musculoskeletal	56	56	56
738.4	Acquired spondylolisthesis	14	musculoskeletal	49	49	49
739	Contracture of joint	14	musculoskeletal	6	6	6
740	Osteoarthrosis	14	musculoskeletal	516	516	516
740.1	Osteoarthritis; localized	14	musculoskeletal	318	318	318
740.11	Osteoarthrosis, localized, primary	14	musculoskeletal	270	270	270
740.12	Osteoarthrosis, localized, secondary	14	musculoskeletal	8	8	8
740.2	Osteoarthrosis, generalized	14	musculoskeletal	72	72	72
740.3	Osteoarthrosis involving more than one site, but not specified as generalized	14	musculoskeletal	10	10	10
740.9	Osteoarthrosis NOS	14	musculoskeletal	196	196	196
741	Symptoms and disorders of the joints	14	musculoskeletal	134	134	134
741.1	Ankylosis of joint	14	musculoskeletal	1	1	1
741.2	Stiffness of joint	14	musculoskeletal	9	9	9
741.3	Difficulty in walking	14	musculoskeletal	4	4	4
741.4	Joint effusions	14	musculoskeletal	70	70	70
741.5	Hemarthrosis	14	musculoskeletal	2	2	2
741.6	Villonodular synovitis	14	musculoskeletal	0	0	0
742	Derangement of joint, non-traumatic	14	musculoskeletal	31	31	31
742.1	Loose body in joint	14	musculoskeletal	0	0	0
742.2	Pathological, developmental or recurrent dislocation	14	musculoskeletal	3	3	3
742.8	Articular cartilage disorder	14	musculoskeletal	8	8	8
742.9	Other derangement of joint	14	musculoskeletal	17	17	17
743	Osteoporosis, osteopenia and pathological fracture	14	musculoskeletal	172	172	172
743.1	Osteoporosis	14	musculoskeletal	34	34	34
743.11	Osteoporosis NOS	14	musculoskeletal	1	1	1
743.12	Senile osteoporosis	14	musculoskeletal	21	21	21
743.13	Other specified osteoporosis	14	musculoskeletal	8	8	8
743.2	Pathologic fracture	14	musculoskeletal	13	13	13
743.21	Pathologic fracture of vertebrae	14	musculoskeletal	8	8	8
743.22	Pathologic fracture of femur	14	musculoskeletal	0	0	0
743.4	Stress fracture	14	musculoskeletal	5	5	5
743.9	Osteopenia or other disorder of bone and cartilage	14	musculoskeletal	16	16	16
745	Pain in joint	14	musculoskeletal	1400	1400	1400
747	Cardiac and circulatory congenital anomalies	15	congenital anomalies	36	36	36
747.1	Cardiac congenital anomalies	15	congenital anomalies	28	28	28
747.11	Cardiac shunt/ heart septal defect	15	congenital anomalies	14	14	14
747.12	Valvular heart disease/ heart chambers	15	congenital anomalies	11	11	11
747.13	Congenital anomalies of great vessels	15	congenital anomalies	3	3	3
747.2	Congenital anomalies of peripheral vascular system	15	congenital anomalies	6	6	6
748	Anomalies of respiratory system, congenital	15	congenital anomalies	0	0	0
749	Congenital anomalies of face and neck	15	congenital anomalies	5	5	5
749.1	Cleft palate	15	congenital anomalies	1	1	1
749.2	Congenital anomalies of skull and face bones	15	congenital anomalies	0	0	0
750	Digestive congenital anomalies	15	congenital anomalies	13	13	13
750.1	Upper gastrointestinal congenital anomalies	15	congenital anomalies	3	3	3
750.11	Esophageal atresia/tracheoesophageal fistula	15	congenital anomalies	3	3	3
750.13	Congenital anomalies of mouth/tongue	15	congenital anomalies	0	0	0
750.14	Congenital anomalies of esophagus	15	congenital anomalies	0	0	0
750.15	Congenital anomalies of stomach	15	congenital anomalies	0	0	0
750.2	Lower gastrointestinal congenital anomalies	15	congenital anomalies	9	9	9
750.21	Congenital anomalies of intestine	15	congenital anomalies	1	1	1
750.22	Congenital anomaly of gallbladder, bile ducts, liver, pancreas	15	congenital anomalies	8	8	8
750.5	Congenital hypertrophic pyloric stenosis	15	congenital anomalies	0	0	0
751	Genitourinary congenital anomalies	15	congenital anomalies	46	46	46
751.1	Congenital anomalies of genital organs	15	congenital anomalies	3	3	3
751.11	Congenital anomalies of female genital organs	15	congenital anomalies	1	1	1
751.12	Congenital anomalies of male genital organs	15	congenital anomalies	1	1	1
751.2	Congenital anomalies of urinary system	15	congenital anomalies	35	35	35
751.21	Cystic kidney disease	15	congenital anomalies	26	26	26
751.22	Other specified congenital anomalies of kidney	15	congenital anomalies	3	3	3

751.3	Obstructive genitourinary defect	15	congenital anomalies	2	2	2
752	Nervous system congenital anomalies	15	congenital anomalies	1	1	1
752.1	Neural tube defects	15	congenital anomalies	0	0	0
752.11	Spina bifida	15	congenital anomalies	0	0	0
752.2	Other specified congenital anomalies of nervous system	15	congenital anomalies	1	1	1
753	Congenital anomalies of the eye	15	congenital anomalies	1	1	1
753.1	Congenital cataract and lens anomalies	15	congenital anomalies	0	0	0
753.2	Congenital anomalies of posterior segment of eye	15	congenital anomalies	0	0	0
754	Congenital musculoskeletal deformities of spine	15	congenital anomalies	6	6	6
754.1	Lumbosacral spondylolysis, congenital	15	congenital anomalies	1	1	1
754.2	Spondylolisthesis, congenital	15	congenital anomalies	3	3	3
755	Congenital anomalies of limbs	15	congenital anomalies	10	10	10
755.1	Congenital deformities of feet	15	congenital anomalies	3	3	3
755.3	Congenital anomaly of fingers/toes	15	congenital anomalies	0	0	0
755.4	Congenital anomalies of upper limb, including shoulder girdle	15	congenital anomalies	1	1	1
755.6	Other congenital anomalies of lower limb, including pelvic girdle	15	congenital anomalies	5	5	5
755.61	Congenital hip dysplasia and deformity	15	congenital anomalies	5	5	5
756	Other congenital musculoskeletal anomalies	15	congenital anomalies	10	10	10
756.1	Congenital anomalies of abdominal wall; diaphram	15	congenital anomalies	0	0	0
756.2	Pectus and other congenital anomalies of ribs/sternum	15	congenital anomalies	4	4	4
756.21	Pectus excavatum	15	congenital anomalies	2	2	2
756.22	Pectus carinatum	15	congenital anomalies	0	0	0
756.3	Congenital anomalies of muscle, tendon, fascia, and connective tissue	15	congenital anomalies	5	5	5
756.5	Congenital osteodystrophies	15	congenital anomalies	0	0	0
757	Congenital anomalies of the integument	15	congenital anomalies	1	1	1
758	Chromosomal anomalies and genetic disorders	15	congenital anomalies	2	2	2
758.1	Chromosomal anomalies	15	congenital anomalies	1	1	1
759	Other and unspecified congenital anomalies	15	congenital anomalies	8	8	8
759.1	Anomalies of endocrine glands, congenital	15	congenital anomalies	2	2	2
760	Back pain	17	symptoms	1036	1036	1036
761	Cervicalgia	17	symptoms	439	439	439
763	Thoracic or lumbosacral neuritis or radiculitis, unspecified	17	symptoms	221	221	221
764	Sciatica	17	symptoms	293	293	293
765	Cervical radiculitis	17	symptoms	98	98	98
766	Neuralgia, neuritis, and radiculitis NOS	17	symptoms	53	53	53
767	Cervicocranial/Cervicobrachial syndrome	17	symptoms	0	0	0
769	Nonallopathic lesions NEC	17	symptoms	28	28	28
770	Myalgia and myositis unspecified	17	symptoms	245	245	245
771	Musculoskeletal symptoms referable to limbs	17	symptoms	25	25	25
771.1	Swelling of limb	17	symptoms	50	50	50
771.2	Cramp of limb	17	symptoms	31	31	31
772	Symptoms of the muscles	17	symptoms	9	9	9
772.1	Muscular wasting and disuse atrophy	17	symptoms	2	2	2
772.2	Spasm of muscle	17	symptoms	132	132	132
772.3	Muscle weakness	17	symptoms	27	27	27
772.4	Rhabdomyolysis	17	symptoms	2	2	2
772.6	Facial weakness	17	symptoms	2	2	2
780	Hypothermia/Chills	17	symptoms	15	15	15
781	Symptoms involving nervous and musculoskeletal systems	17	symptoms	113	113	113
781.1	Loss of height	17	symptoms	2	2	2
781.2	Abnormal posture	17	symptoms	0	0	0
782	Symptoms involving skin and other integumentary tissue	17	symptoms	236	236	236
782.3	Edema	17	symptoms	191	191	191
782.6	Pallor and flushing	17	symptoms	27	27	27
783	Fever of unknown origin	17	symptoms	234	234	234
783.1	Postprocedural fever	17	symptoms	2	2	2
785	Abdominal pain	17	symptoms	929	929	929
788	Syncope and collapse	17	symptoms	209	209	209
789	Nausea and vomiting	17	symptoms	544	544	544
789.1	Persistent vomiting	17	symptoms	9	9	9
790	Nonspecific findings on examination of blood	17	symptoms	18	18	18
790.1	Elevated sedimentation rate	17	symptoms	10	10	10
790.6	Other abnormal blood chemistry	17	symptoms	125	125	125
790.8	Elevated C-reactive protein (CRP)	17	symptoms	16	16	16
790.9	Abnormal arterial blood gases	17	symptoms	2	2	2
792	Abnormal Papanicolaou smear of cervix and cervical HPV	17	symptoms	40	40	40
792.1	Papanicolaou smear of cervix or vagina with atypical squamous cells	17	symptoms	30	30	30

794	Abnormal results of other function studies (bladder, pancreas, placenta, spleen, etc)	17	symptoms	0	0	0
795	Other and nonspecific abnormal cytological, histological and immunological findings	17	symptoms	8	8	8
795.8	Abnormal tumor markers	17	symptoms	2	2	2
795.81	Elevated carcinoembryonic antigen [CEA]	17	symptoms	1	1	1
795.82	Elevated cancer antigen 125 [CA 125]	17	symptoms	1	1	1
796	Elevated prostate specific antigen [PSA]	17	symptoms	64	64	64
797	Shock	17	symptoms	7	7	7
797.1	Cardiogenic shock	17	symptoms	0	0	0
798	Malaise and fatigue	17	symptoms	1164	1164	1164
798.1	Chronic fatigue syndrome	17	symptoms	19	19	19
800	Fracture of lower limb	18	injuries & poisonings	56	56	56
800.1	Fracture of neck of femur	18	injuries & poisonings	18	18	18
800.2	Fracture of unspecified part of femur	18	injuries & poisonings	2	2	2
800.3	Fracture of tibia and fibula	18	injuries & poisonings	21	21	21
800.4	Fracture of patella	18	injuries & poisonings	9	9	9
801	Fracture of ankle and foot	18	injuries & poisonings	95	95	95
801.1	Fracture of foot	18	injuries & poisonings	32	32	32
802	Fracture of pelvis	18	injuries & poisonings	6	6	6
803	Fracture of upper limb	18	injuries & poisonings	91	91	91
803.1	Fracture of humerus	18	injuries & poisonings	26	26	26
803.2	Fracture of radius and ulna	18	injuries & poisonings	45	45	45
803.21	Colles' fracture	18	injuries & poisonings	3	3	3
803.3	Fracture of clavicle or scapula	18	injuries & poisonings	14	14	14
804	Fracture of hand or wrist	18	injuries & poisonings	38	38	38
805	Fracture of vertebral column without mention of spinal cord injury	18	injuries & poisonings	49	49	49
807	Fracture of ribs	18	injuries & poisonings	36	36	36
809	Fracture of unspecified bones	18	injuries & poisonings	21	21	21
816	Cerebral laceration and contusion	18	injuries & poisonings	0	0	0
817	Concussion	18	injuries & poisonings	26	26	26
818	Intracranial hemorrhage (injury)	18	injuries & poisonings	8	8	8
818.1	Subdural hemorrhage (injury)	18	injuries & poisonings	5	5	5
818.2	Subarachnoid hemorrhage (injury)	18	injuries & poisonings	0	0	0
819	Skull and face fracture and other intercranial injury	18	injuries & poisonings	41	41	41
823	Fracture of tibia and fibula	18	injuries & poisonings	0	0	0
830	Dislocation	18	injuries & poisonings	120	120	120
835	Internal derangement of knee	18	injuries & poisonings	93	93	93
836	Traumatic arthropathy	18	injuries & poisonings	2	2	2
840	Sprains and strains	18	injuries & poisonings	445	445	445
840.1	Muscle/tendon sprain	18	injuries & poisonings	10	10	10
840.2	Rotator cuff (capsule) sprain	18	injuries & poisonings	15	15	15
840.3	Joint/ligament sprain	18	injuries & poisonings	34	34	34
841	Sprains and strains of back and neck	18	injuries & poisonings	257	257	257
842	Other sprains and strains	18	injuries & poisonings	51	51	51
850	Hemorrhage or hematoma complicating a procedure	18	injuries & poisonings	9	9	9
851	Complications of transplants and reattached limbs	18	injuries & poisonings	0	0	0
853	Complication of colostomy or enterostomy	18	injuries & poisonings	4	4	4
854	Complications of cardiac/vascular device, implant, and graft	18	injuries & poisonings	2	2	2
855	Complication of nervous system device, implant, and graft	18	injuries & poisonings	0	0	0
856	Vascular complications of surgery and medical procedures	18	injuries & poisonings	0	0	0
857	Mechanical complication of unspecified genitourinary device, implant, and graft	18	injuries & poisonings	6	6	6
858	Complication of internal orthopedic device	18	injuries & poisonings	16	16	16
859	Complication due to other implant and internal device	18	injuries & poisonings	11	11	11
860	Bone marrow or stem cell transplant	2	neoplasms	2	2	2
870	Open wounds of head; neck; and trunk	18	injuries & poisonings	121	121	121
870.1	Open wound or laceration of eye or eyelid	18	injuries & poisonings	2	2	2
870.2	Open wound of ear	18	injuries & poisonings	0	0	0
870.3	Other open wound of head and face	18	injuries & poisonings	32	32	32
870.4	Open wound of nose and sinus	18	injuries & poisonings	3	3	3
870.5	Open wound of lip and mouth	18	injuries & poisonings	9	9	9
870.6	Open wound of neck	18	injuries & poisonings	1	1	1
870.8	Open wound of genital organs	18	injuries & poisonings	2	2	2
871	Open wounds of extremities	18	injuries & poisonings	243	243	243
871.1	Open wound of hand except finger(s)	18	injuries & poisonings	50	50	50
871.2	Open wound of finger(s)	18	injuries & poisonings	112	112	112
871.3	Open wound of foot except toe(s) alone	18	injuries & poisonings	14	14	14
871.4	Open wound of toe(s)	18	injuries & poisonings	16	16	16
872	Traumatic amputation	18	injuries & poisonings	4	4	4

874	Complication of amputation stump	18	injuries & poisonings	0	0	0
875	Non-healing surgical wound	18	injuries & poisonings	2	2	2
876	Posttraumatic wound infection not elsewhere classified	18	injuries & poisonings	43	43	43
907	Injuries to the nervous system	18	injuries & poisonings	8	8	8
910	Superficial injury, infected	18	injuries & poisonings	17	17	17
911	Blister	18	injuries & poisonings	8	8	8
912	Insect bite	18	injuries & poisonings	38	38	38
913	Toxic effect of venom	18	injuries & poisonings	16	16	16
916	Contusion	18	injuries & poisonings	295	295	295
930	Allergic reaction to food	18	injuries & poisonings	38	38	38
931	Contact dermatitis and other eczema due to plants [except food]	18	injuries & poisonings	1	1	1
938	Dermatitis due to solar radiation	18	injuries & poisonings	16	16	16
938.1	Acute dermatitis due to solar radiation	18	injuries & poisonings	8	8	8
938.2	Chronic dermatitis due to solar radiation	18	injuries & poisonings	1	1	1
939	Atopic/contact dermatitis due to other or unspecified	13	dermatologic	246	246	246
939.1	Contact and allergic dermatitis of eyelid	13	dermatologic	3	3	3
941	Adverse reaction to serum or vaccine	18	injuries & poisonings	1	1	1
942	Infusion and transfusion reaction	18	injuries & poisonings	1	1	1
946	Anaphylactic shock NOS	18	injuries & poisonings	10	10	10
947	Urticaria	18	injuries & poisonings	75	75	75
949	Allergies, other	18	injuries & poisonings	216	216	216
949.1	Diaper or napkin rash	18	injuries & poisonings	1	1	1
952	Spinal cord injury without evidence of spinal bone injury	18	injuries & poisonings	2	2	2
957	Injury to other and unspecified nerves	18	injuries & poisonings	0	0	0
958	Certain early complications of trauma or procedure	18	injuries & poisonings	4	4	4
958.1	Postoperative shock	18	injuries & poisonings	1	1	1
958.2	Traumatic and surgical subcutaneous emphysema	18	injuries & poisonings	0	0	0
960	Poisoning by antibiotics	18	injuries & poisonings	130	130	130
960.1	Adverse effects of antibacterials (not penicillins)	18	injuries & poisonings	0	0	0
960.2	Allergy/adverse effect of penicillin	18	injuries & poisonings	55	55	55
960.3	Poisoning by antifungal antibiotics	18	injuries & poisonings	0	0	0
961	Poisoning by other anti-infectives	18	injuries & poisonings	1	1	1
961.1	Poisoning/allergy of sulfonamides	18	injuries & poisonings	36	36	36
962	Poisoning by hormones and synthetic substitutes	18	injuries & poisonings	12	12	12
962.1	Adrenal cortical steroids causing adverse effects in therapeutic use	18	injuries & poisonings	9	9	9
962.2	Insulins and antidiabetic agents causing adverse effects in therapeutic use	18	injuries & poisonings	2	2	2
962.3	Hormones and synthetic substitutes causing adverse effects in therapeutic use	18	injuries & poisonings	1	1	1
963	Poisoning by primarily systemic agents	18	injuries & poisonings	17	17	17
963.1	Antineoplastic and immunosuppressive drugs causing adverse effects	18	injuries & poisonings	15	15	15
964	Poisoning by agents primarily affecting blood constituents	18	injuries & poisonings	1	1	1
964.1	Anticoagulants causing adverse effects	18	injuries & poisonings	1	1	1
965	Poisoning by analgesics, antipyretics, and antirheumatics	18	injuries & poisonings	13	13	13
965.1	Opiates and related narcotics causing adverse effects in therapeutic use	18	injuries & poisonings	41	41	41
965.2	Antirheumatics causing adverse effects in therapeutic use	18	injuries & poisonings	0	0	0
965.3	Salicylates causing adverse effects in therapeutic use	18	injuries & poisonings	0	0	0
966	Poisoning by anticonvulsants and anti-Parkinsonism drugs	18	injuries & poisonings	0	0	0
967	Adverse effects of sedatives or other central nervous system depressants and anesthetics	18	injuries & poisonings	4	4	4
969	Poisoning by psychotropic agents	18	injuries & poisonings	4	4	4
971	Poisoning by drugs primarily affecting the autonomic nervous system	18	injuries & poisonings	3	3	3
972	Poisoning by agents primarily affecting the cardiovascular system	18	injuries & poisonings	19	19	19
972.1	Cardiac rhythm regulators causing adverse effects in therapeutic use	18	injuries & poisonings	1	1	1
972.2	Antilipemic and antiarteriosclerotic drugs causing adverse effects in therapeutic use	18	injuries & poisonings	3	3	3
972.6	Antihypertensive agents causing adverse effects	18	injuries & poisonings	15	15	15
973	Poisoning by agents primarily affecting the gastrointestinal system	18	injuries & poisonings	0	0	0
974	Poisoning by water, mineral, and uric acid metabolism drugs	18	injuries & poisonings	4	4	4
975	Poisoning by agents primarily acting on the smooth and skeletal muscles and respiratory system	18	injuries & poisonings	2	2	2
976	Poisoning by agents primarily affecting skin & mucous membrane, ophthalmological, otorhinolaryngological, & dental drugs	18	injuries & poisonings	1	1	1
977	Personal history of allergy to medicinal agents	18	injuries & poisonings	18	18	18
979	Adverse drug events and drug allergies	18	injuries & poisonings	49	49	49
980	Encounter for long-term (current) use of antibiotics	1	infectious diseases	2	2	2
981	Toxic effect of (non-ethyl) alcohol and petroleum and other solvents	18	injuries & poisonings	0	0	0
983	Toxic effect of corrosive aromatics, acids, and caustic alkalis	18	injuries & poisonings	2	2	2
984	Toxic effect of lead and its compounds (including fumes)	18	injuries & poisonings	0	0	0
985	Toxic effect of other metals	18	injuries & poisonings	1	1	1
986	Toxic effect of carbon monoxide	18	injuries & poisonings	0	0	0
987	Toxic effect of other gases, fumes, or vapors	18	injuries & poisonings	1	1	1
988	Toxic effect of noxious substances eaten as food	18	injuries & poisonings	1	1	1

989	Toxic effect of other substances, chiefly nonmedicinal as to source	18	injuries & poisonings	1	1	1
990	Effects radiation NOS	18	injuries & poisonings	6	6	6
994	Sepsis and SIRS	18	injuries & poisonings	65	65	65
994.1	Systemic inflammatory response syndrome (SIRS)	18	injuries & poisonings	1	1	1
994.2	Sepsis	18	injuries & poisonings	55	55	55
994.21	Septic shock	18	injuries & poisonings	16	16	16
