# Supplementary figures and images for: GWAS and PheWAS of red blood cell components in a Northern Nevadan cohort
Source: PLoS One. 2019 Jun 13;14(6):e0218078. doi: 10.1371/journal.pone.0218078 (PMC6564422; doi:10.1371/journal.pone.0218078)

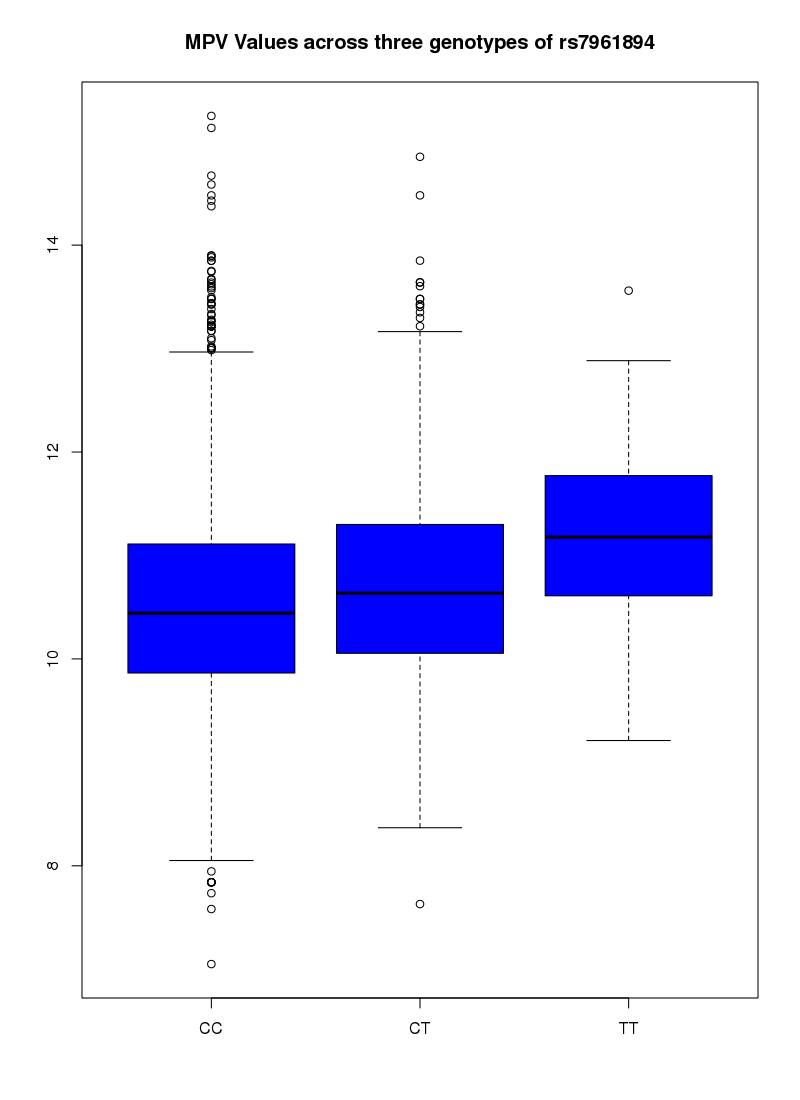

Supplement: S1 Fig — (A, B, C): GWAS results for RBC components MPV, MCV and PC. Genome-wide association study results for the three RBC components. The x-axis represents the genomic position of 498,916 SNPs. The y-axis represents -log10-transformed raw p-values of each genotypic association. The red horizontal line indicates the threshold of significance p = 5x10^-8^. (TIFF) [file pone.0218078.s004.tiff]

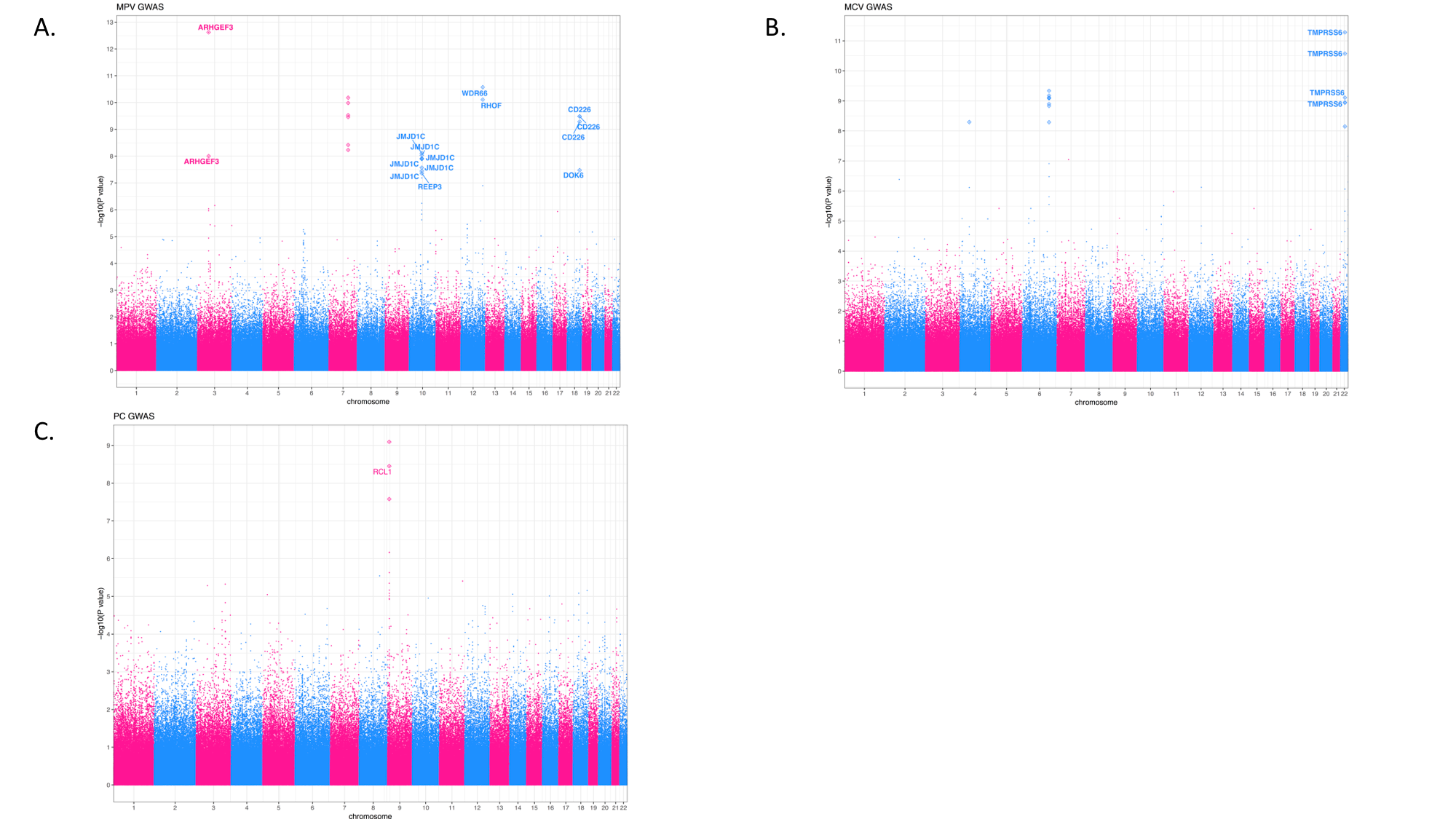

Supplement: S2 Fig — This figure shows the box and whisker diagram for standardized values of MPV of all members in the cohort based on genotype. Mean and standard deviation values for each genotype are CC: 10.54 ± 0.97; CT: 10.74 ± 1.0; TT: 11.21 ± 0.87. The p-value for this ANOVA analysis is p = 8.7x10^-12^. (TIFF) [file pone.0218078.s005.tiff]

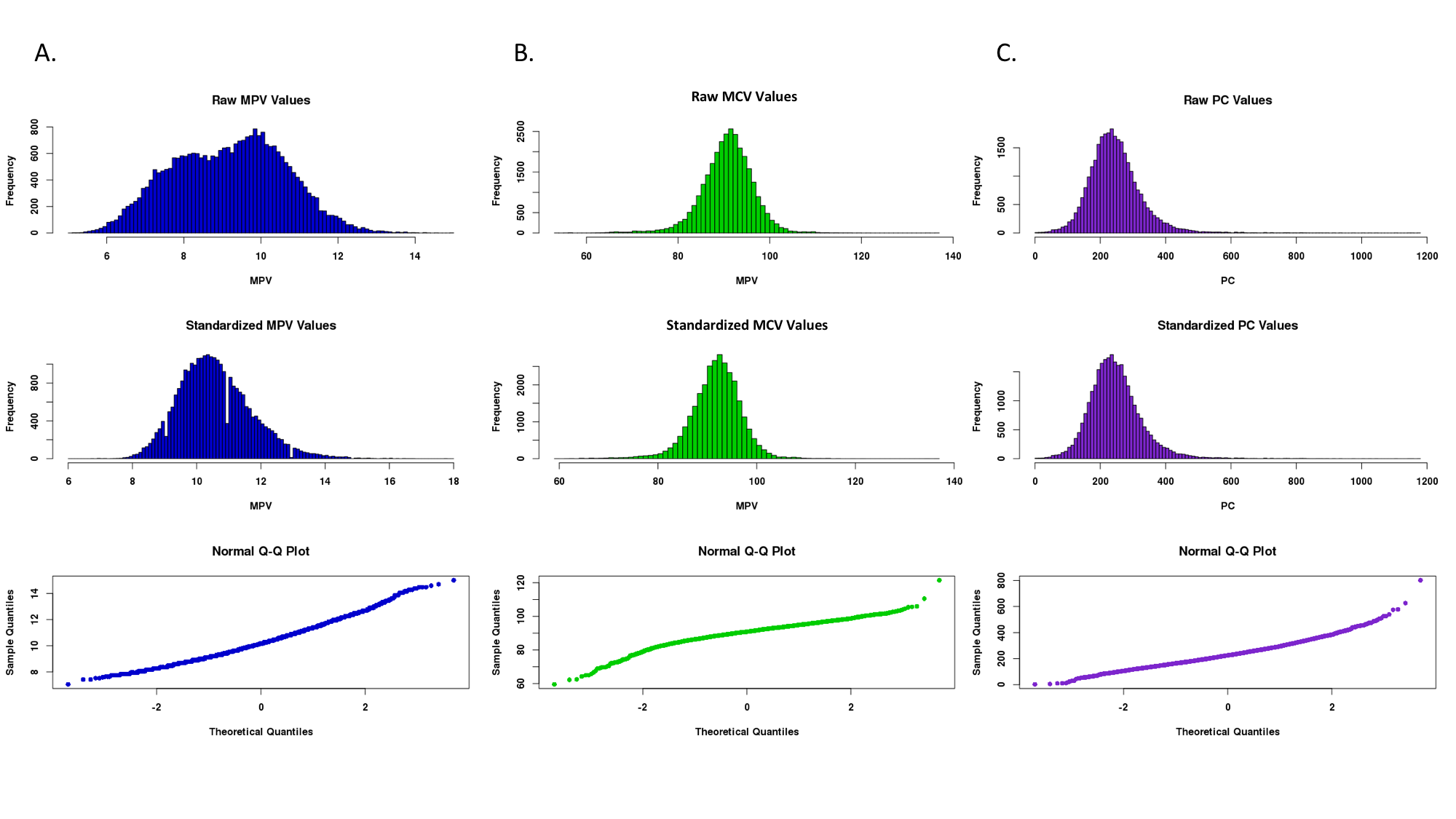

Supplement: S3 Fig — (A, B, C): PheWAS results between RBC component-significant SNPs and phecodes. These three figures show the results of individual logistic regressions between incidence of phenotype groups (phecodes) and SNP genotypes, based on the additive model. Models include age, gender and ethnicity as covariates. Each point represents the p-value of one SNP and one of 1,488 phecodes with at least 20 cases assigned to it. The horizontal red line in each represents the significance level p = 1.60x10^-6^ for MPV, p = 2.40x10^-6^ for MCV, and p = 1.12x10^-5^ for PC. (TIFF) [file pone.0218078.s006.tiff]

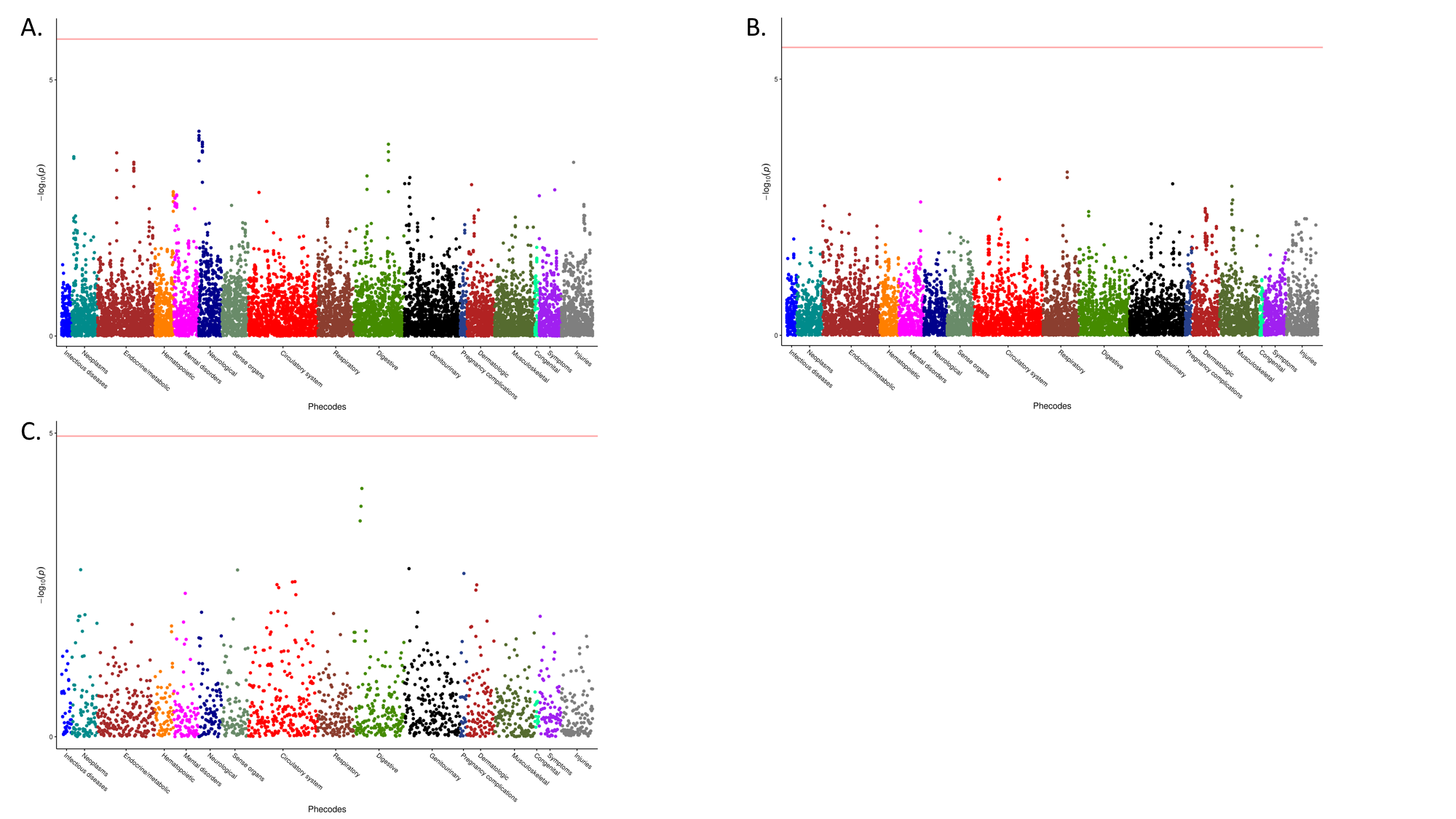

Supplement: S4 Fig — (A, B, C): PheWAS results between RBC component and phecodes. These three figures show the results of individual linear regressions between incidence of phenotype groups (phecodes) and continuous RBC component measures. Models include age, gender and ethnicity as covariates. Each point represents the p-value of the association between one of 1,488 phecodes with at least 20 cases assigned to it, and the RBC component measure. The horizontal red line in each represents the significance level p = 1.60x10^-6^ for MPV, p = 2.40x10^-6^ for MCV, and p = 1.12x10^-5^ for PC. (TIFF) [file pone.0218078.s007.tiff]

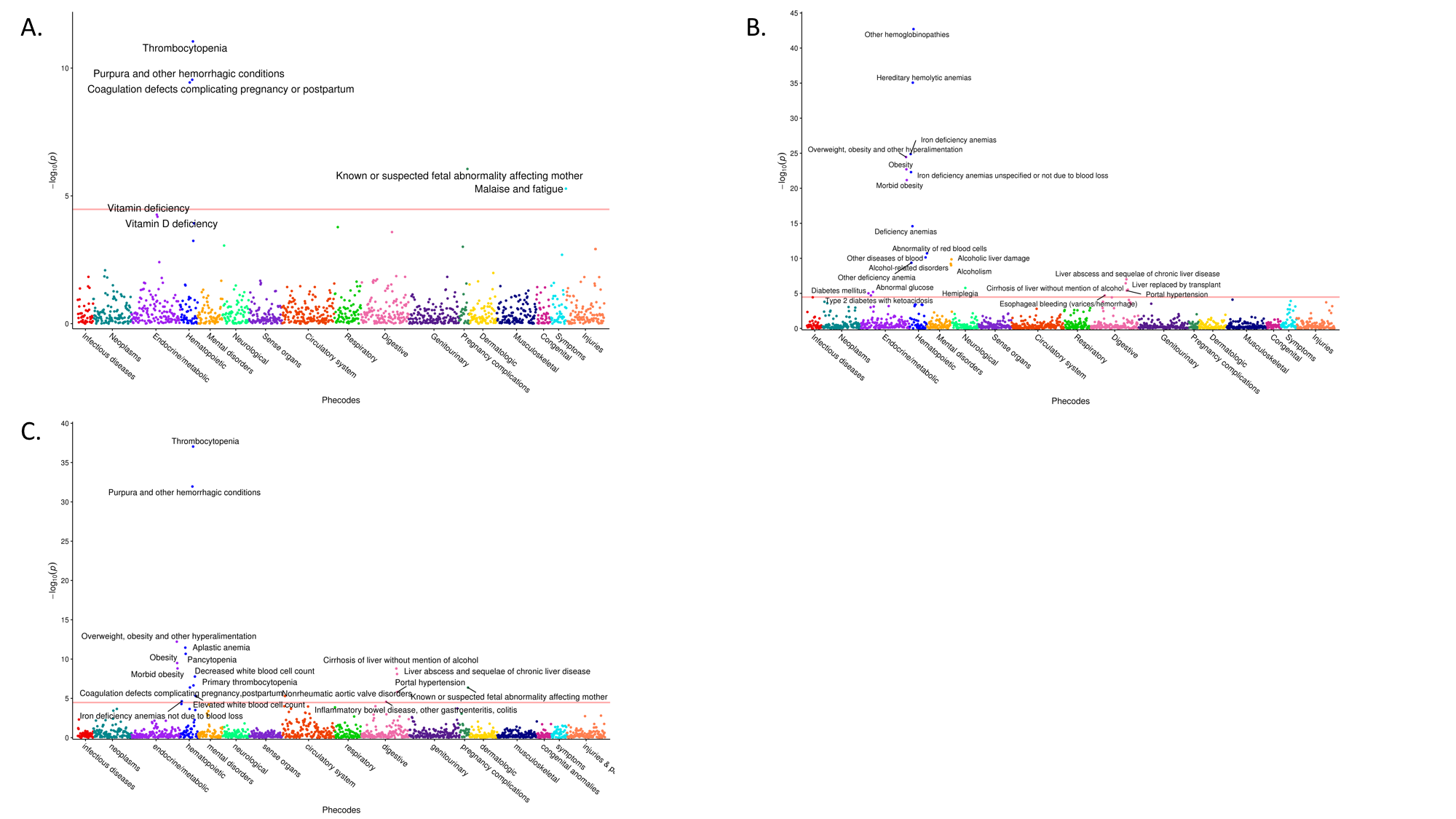

Supplement: S5 Fig — (A, B, C): Raw and standardized RBC component lab measures. Distribution of raw RBC component values are presented in the first row; distribution of component values upon standardization to the most recent lab test are shown in the second row; the QQ-plot of the standardized values is pictured in the third row. (TIFF) [file pone.0218078.s008.tiff]
